# Human NK cells confer protection against HIV-1 infection in humanized mice

**DOI:** 10.1172/JCI162694

**Published:** 2022-12-15

**Authors:** Can M. Sungur, Qiankun Wang, Ayşe N. Ozantürk, Hongbo Gao, Aaron J. Schmitz, Marina Cella, Wayne M. Yokoyama, Liang Shan

**Affiliations:** 1 Division of Rheumatology, Department of Medicine,; 2 Division of Infectious Diseases, Department of Medicine,; 3 Department of Pathology and Immunology, and; 4 The Andrew M. and Jane M. Bursky Center for Human Immunology and Immunotherapy Programs, Washington University School of Medicine, Saint Louis, Missouri, USA.

**Keywords:** Immunology, NK cells

## Abstract

The role of NK cells against HIV-1 infections remains to be elucidated in vivo. While humanized mouse models potentially could be used to directly evaluate human NK cell responses during HIV-1 infection, improved functional development of human NK cells in these hosts is needed. Here, we report the humanized MISTRG-6-15 mouse model, in which NK cells were quick to expand and exhibit degranulation, cytotoxicity, and proinflammatory cytokine production in nonlymphoid organs upon HIV-1 infection but had reduced functionality in lymphoid organs. Although HIV-1 infection induced functional impairment of NK cells, antiretroviral therapy reinvigorated NK cells in response to HIV-1 rebound after analytic treatment interruption. Moreover, a broadly neutralizing antibody, PGT121, enhanced NK cell function in vivo, consistent with antibody-dependent cellular cytotoxicity. Monoclonal antibody depletion of NK cells resulted in higher viral loads in multiple nonlymphoid organs. Overall, our results in humanized MISTRG-6-15 mice demonstrated that NK cells provided direct anti–HIV-1 responses in vivo but were limited in their responses in lymphoid organs.

## Introduction

NK cells recognize ligands presented on the surface of HIV-1–infected cells, including infection-induced self-proteins (1, 2) and possibly the viral envelope protein (3), as well as Fc regions of viral-specific antibodies (4, 5) and MHC class I molecules (6, 7) that engage activating or inhibitory NK receptors, respectively. Genetic and epidemiological studies strongly support an active role for NK cells in HIV-1 infection. Interestingly, the combined genotype of certain germline-encoded killer immunoglobulin-like receptors (KIRs) — generally selectively expressed on NK cells — and their HLA ligands are associated with slower HIV-1 disease progression (8, 9) or protection against HIV-1 acquisition (10–12). KIR-dependent NK cell activities appear to directly mediate immune pressure leading to HIV-1 evolution in infected individuals (13). In addition, antibody-dependent cellular cytotoxicity (ADCC), potentially mediated by NK cells, is associated with HIV-1 control (14–17) and is linked to vaccine-induced protective immunity against HIV infection (18–20). NK cells can inhibit HIV replication but these studies have been limited to in vitro and ex vivo studies on NK cells from peripheral blood samples (21–25). Thus, the in vivo role of NK cells in direct control of HIV-1 infections has not been elucidated.

In mouse experimental models, NK cells have disparate effector functions across tissues, and tissue-resident NK cells acquire unique functions different from blood NK cells for viral containment (26, 27). In humans, evidence for discrete stages of differentiation and functional development of blood and tissue NK cells has also been reported. While most NK cells in the blood are mature (CD56^dim^CD16^+^) with a strong capacity to lyse target cells, the immature and noncytolytic CD56^bright^CD16^–^ NK cells are the predominant subset in secondary lymphoid tissues (28–30), which are the major sites for HIV-1 infection in untreated individuals (31–33) and the most important anatomical reservoirs for latent HIV-1 in people on suppressive antiretroviral therapy (ART) (34). However, there is a relative scarcity of studies assessing the pleiotropic functions of tissue NK cells during HIV-1 infection due to a lack of easy-access tissue samples. Therefore, how NK cells influence HIV-1 infection in vivo remains unknown, despite strong evidence from epidemiological and in vitro studies.

Generation of human immune system mice (humanized mice) can be achieved in various strains of immunodeficient mice transplanted with human hematopoietic stem and progenitor cells. Humanized mice are useful tools to study HIV-1 infection and immune responses (35) but human NK cells reconstituted in immunodeficient mice are numerically and functionally deficient, secrete lower levels of IFN-γ and do not respond to NK cell–susceptible targets (36, 37). This may be due to the lack of cross reactivity between murine IL-15 and human IL-15 receptors, since IL-15 is essential for the development and survival of NK cells and human IL-15 injection can rescue differentiation of human NK cells (38, 39). However, IL-15 supplementation is only temporary, and the appropriate distribution of IL-15–expressing cells that is critical for development and maintenance of NK cells with circulating or tissue-resident identities (40) cannot be achieved through cytokine injection or transgenic expression. Both hematopoietic and nonhematopoietic cells provide IL-15 and IL-15 receptor α (IL-15Rα) and both can drive NK cell differentiation with hematopoietic-derived IL-15Rα being more important to the late maturation process (41). MISTRG mice with knock-in expression of human M-CSF, IL-3/GM-CSF, SIRPα, and THPO that together support efficient development of human myeloid cells — the hematopoietic source of IL-15 — showed improved development of human NK cells in tissues (42). Since human IL-15 production in the MISTRG model is solely dependent on human myeloid cells, experimental conditions that destroy the myeloid cell compartment, such as clodronate or HIV-1 infection, lead to rapid loss of NK cells (42, 43). On the other hand, a mouse with human *IL15* and signal regulatory protein α (*SIRPA*) knockin, named SRG-15, can provide human IL-15 from the nonhematopoietic compartment comprising murine stromal cells and epithelial cells, as it does not support efficient human myelopoiesis (40). SRG-15 mice promote improved human NK cell maturation in the bone marrow and periphery, which rely on the suboptimal cross-reactivity between human IL-15 and murine IL-15RA (44). To further improve NK cell development, we generated MISTRG mice with humanized IL-6 and IL-15, termed MISTRG-6-15, which produce human IL-15 from both hematopoietic and nonhematopoietic cell compartments, which is more physiologically relevant than the MISTRG and SRG-15 models. Since IL-6 stimulates hematopoietic progenitor cells and myeloid differentiation in mice (45), IL-6 humanization partially blocks murine hematopoiesis to better support overall reconstitution of multiple lineages of human immune cells.

Here, we characterized human NK cell development and its functional dynamics during acute and chronic HIV-1 infection or after ART in humanized MISTRG-6-15 mice. More importantly, we showed that NK cell depletion led to increased HIV-1 replication and accelerated disease progression in vivo. Thus, we provide direct evidence that human NK cells can control HIV-1 infection in vivo.

## Results

### Functional development of human NK cells in humanized MISTRG-6-15 mice.

**Figure 1:**
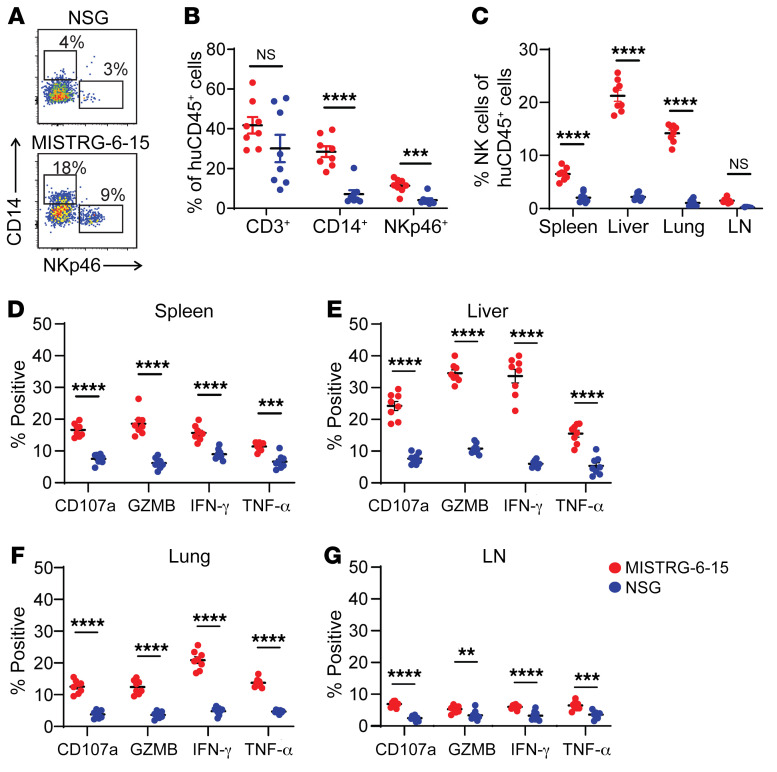
Improved human NK cell reconstitution and functionality in humanized MISTRG-6-15 versus NSG mice. (**A**) Representative flow cytometry plots of NKp46^+^ and CD14^+^ cells in blood between MISTRG-6-15 and NSG mice. (**B**) Percentage of CD3^+^, CD14^+^, and NKp46^+^ cells in blood. Cells were gated on human CD45^+^ population. (**C**) Percentage of CD3^–^CD56^+^ NK cells (defined by CD56 and not NKp46, to reduce inclusion of group 1 and 3 innate lymphoid cells) in spleen, liver, lung, and LN. Cells were gated on human CD45^+^ population. (**D**–**F**) Percentage of CD107a^+^, GZMB^+^, IFNγ^+^, and TNFα^+^ NK cells in spleen (**D**), liver (**E**), lung (**F**), and LN (**G**) after 4 hours of ex vivo stimulation with PMA/ionomycin. Data displayed as mean ± SEM. 8 mice per group. *P* values were calculated using 2-way ANOVA with Šidák’s multiple comparison test. **P < 0.01, ***P < 0.001, and ****P < 0.0001.

We first compared reconstitution and functionality of human NK cells in reconstituted MISTRG-6-15 and the commonly used NSG mice. MISTRG-6-15 mice had significantly more circulating NK cells and monocytes compared with NSG mice engrafted with the same cord blood sample (Figure 1, A and B). In addition, tissue NK cells — which are defined by CD56 and not NKp46 to reduce the inclusion of group 1 and 3 innate lymphoid cells — were also significantly higher in MISTRG-6-15 mice (Figure 1C and Supplemental Figure 1; supplemental material available online with this article; https://doi.org/10.1172/JCI162694DS1). Furthermore, NK cells in all tissues of the MISTRG-6-15 mice produced more cytokines (Figure 1, D–G) and had stronger cytolytic capacity (Supplemental Figure 2A) than those in NSG mice. Concanamycin A was used to confirm that the killing of K562 cells was perforin dependent (Supplemental Figure 2B). Thus, human NK cells show improved reconstitution in both number and function in MISTRG-6-15 mice compared with NSG mice.

**Figure 2:**
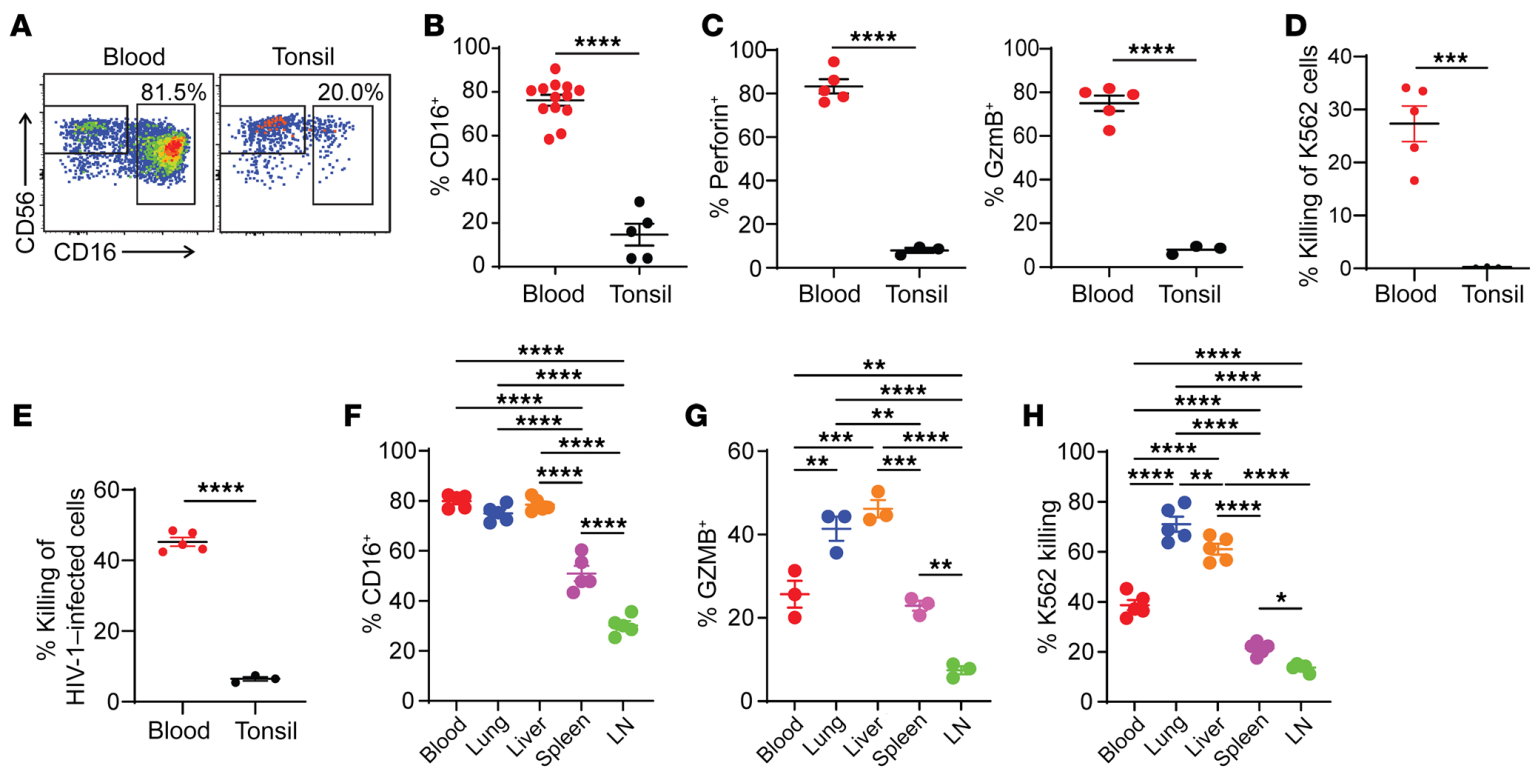
Lymphoid tissue NK cells isolated from healthy human donors are functionally defective against HIV-1–infected cells. (**A–E**) NK cells from human blood and tonsils were gated on CD3^–^CD56^+^ population. (**A**) Representative flow cytometry plots of CD56 and CD16 expression of human blood and tonsil NK cells. (**B**) Percentage of CD16^+^ NK cells in blood (*n* = 13) and tonsil (*n* = 5). (**C**) Percentage of Perforin^+^ or GZMB^+^ NK cells in blood and tonsil after 4 hours of PMA/ionomycin stimulation. (**D** and **E**) Percentage of killing of K562 (**D**) and HIV-1 infected CD4^+^ T cells (**E**) after coculture for 4 hours at 1:1 effector-to-target ratio (E:T). Symbols represent biologically independent samples isolated from blood (*n* = 5) and tonsils (*n* = 3) from healthy donors. (**F**–**H**) in humanized MISTRG-6-15 mice, percentage of (**F**) CD16^+^ NK cells (*n* = 5), (**G**) GZMB^+^ NK cells (*n* = 3), and (**H**) K562 killing (*n* = 5) by NK cells in the blood, lung, liver, spleen, and LN after ex vivo culture for 4 hours with K562 targets at 1:1 E:T. Data displayed as mean ± SEM. In **B**–**E**, *P* values were calculated using unpaired, 2-tailed *t* tests. In **F**–**H**, *P* values were calculated using 1-way ANOVA with Tukey’s multiple comparison post test. **P < 0.01, ***P < 0.001, and ****P < 0.0001.

Human NK cell functions vary widely across tissues, particularly with respect to lymphoid NK cells. Indeed, the frequency of NK cells in lymph nodes (LNs) of MISTRG-6-15 mice was low, and the vast majority of LN NK cells (over 90%) were functionally immature (Figure 1G) compared with NK cells in the spleen, liver, and lung (Figure 1, D–F). To further compare functions of lymphoid NK cells from humans and MISTRG-6-15 mice, we purified NK cells from human tonsil and blood samples. Similar to previous studies (28–30), we found that the frequency of NK cells expressing CD16, granzyme B (GZMB), or perforin was significantly higher in blood compared with tonsil (Figure 2, A–C), and tonsil NK cells could not lyse K562 cells (Figure 2D) or autologous CD4^+^ T cells infected by HIV-1 (Figure 2E). The predominance of immature NK cells in lymphoid tissues is likely due to their expression of the homing receptor CCR7, which was virtually absent in mature CD16^+^ NK cells (Supplemental Figure 3). To evaluate the ADCC activity, we cocultured blood and tonsil NK cells with autologous CD4^+^ T cells infected with a recombinant HIV-1 reporter virus (HIVivo-HA) (Supplemental Figure 4). The humanized IgG1 anti-HA antibody could bind to HIVivo-HA infected cells but had no neutralizing activity (46). Blood NK cells were able to lyse more than 40% of the infected autologous blood CD4^+^ T cells in a Fc-dependent manner, as evidenced by the complete loss of cell lysis when treated with a mutant anti-HA antibody carrying mutations (GRLR) that abrogated binding to activating Fc receptors. In contrast, tonsil NK cells exhibited minimal cytolysis of the autologous tonsil CD4^+^ T cells regardless of treatment, similar to controls without antibody. Similar to the disparate tissue distribution patterns in humans, the vast majority of NK cells in blood and nonlymphoid tissues of MISTRG-6-15 mice exhibited mature phenotypes with CD16 expression (Figure 2F and Supplemental Figure 5). By contrast, the dominant presence of CD16^–^ immature NK cells was observed in LNs of MISTRG-6-15 mice (Figure 2F). These NK cells produced very low levels of effector molecules (Figure 2G) and were unable to kill K562 cells (Figure 2H). Overall, the functional development of human NK cells in MISTRG-6-15 mice with tissue-specific disparities comparable to humans allowed us to compare tissue-specific NK cell responses with HIV-1 infection.

### Dynamics of NK cell responses in MISTRG-6-15 mice during acute and chronic HIV-1 infection.

**Figure 3:**
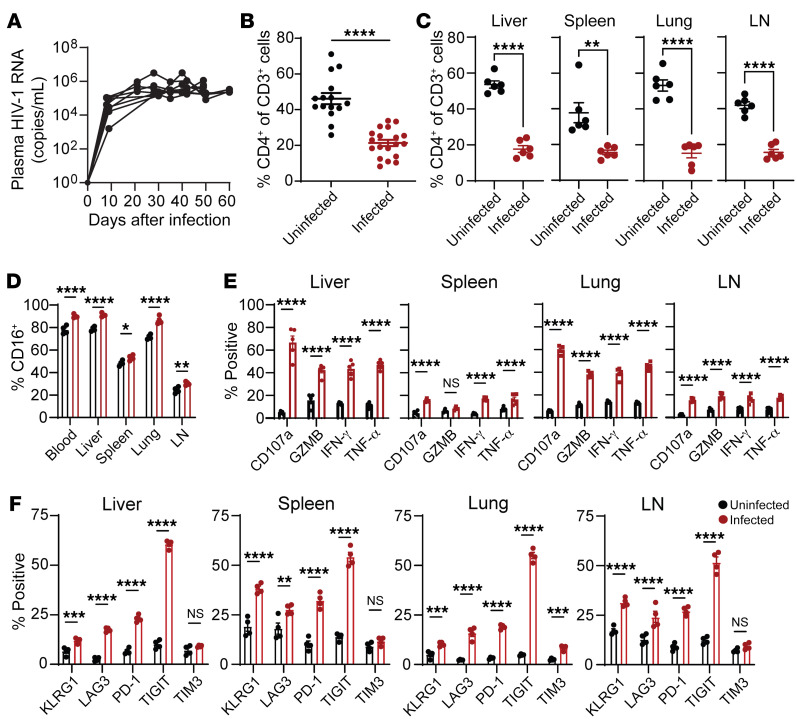
Human NK cells respond to HIV-1 infection in humanized MISTRG-6-15 mice. MISTRG-6-15 mice were infected with HIV-1_BAL_. (**A**) Longitudinal viral load measurement (*n* = 8). Lines connect data from the same mice. For analysis in **B**–**F**, blood and tissue samples were collected on day 21 after infection. (**B** and **C**) Percent CD4^+^ of total T cells in blood (**B**) (*n* = 15) or tissues (**C**) (*n* = 6) of uninfected and infected MISTRG-6-15 mice. Cells were gated on human CD45^+^CD3^+^ population. (**D**) Percentage of blood and tissue NK cells positive for CD16. (**E**) Percentage of tissue NK cells positive for CD107a, GZMB, IFN-γ, and TNF-α after ex vivo stimulation with PMA/ionomycin for 4 hours. In **D** and **E**, 5 mice were used per group. (**F**) Percentage of tissue NK cells positive for KLRG1, LAG3, PD-1, TIGIT, and TIM3. In **D** and **E**, 5 mice were used per group. In **F**, 4 mice were used per group. Data displayed as mean ± SEM. In **B** and **C**, *P* values were calculated using unpaired, 2-tailed *t* tests. In **E** and **F**, *P* values were calculated using 2-way ANOVA with Šidák’s multiple comparison test. *P < 0.05,**P < 0.01, ***P < 0.001, and ****P < 0.0001.

**Figure 4:**
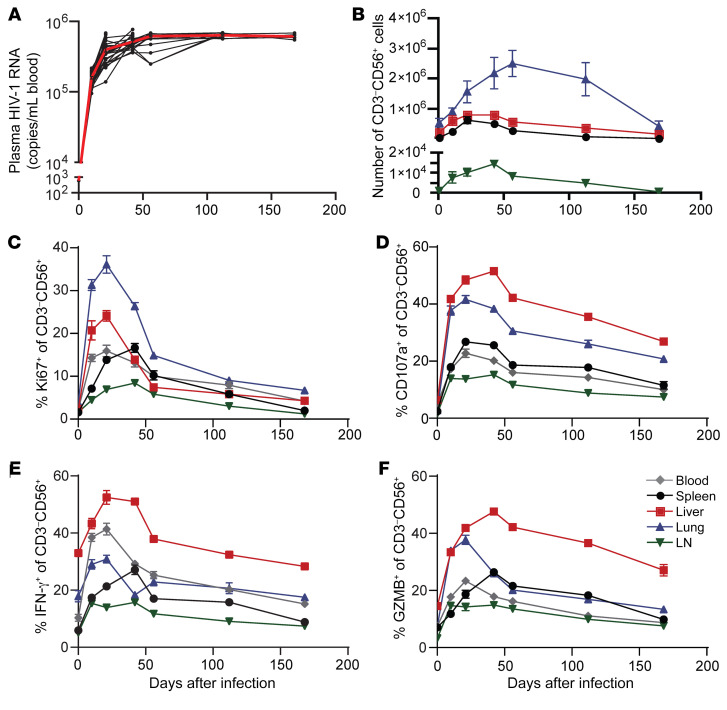
NK cell expansion and increased functionality during the course of HIV-1 infection wanes with time. MISTRG-6-15 mice were infected with HIV-1_BAL_. On days 0, 10, 21, 42, 56, 112, and 168 after infection, 4–6 mice at each time point were euthanized for blood and tissue collection. (**A**) Plasma HIV-1 RNA levels; red line illustrates the average. (**B**) Number of human NK cells (huCD45^+^CD3^–^CD56^+^) throughout the course of infection in spleen, liver, lung, and LN. (**C**–**F**) Ki67, CD107a, IFN-γ, and GZMB expression by NK cells in blood, spleen, liver, lung, and LN. NK cells purified from tissues were stimulated with PMA/ionomycin ex vivo for 4 hours before flow cytometry analysis. Data displayed as mean ± SEM.

We infected MISTRG-6-15 with HIV-1_BaL_ strain to characterize viral infection. Plasma HIV-1 RNA was readily detectable as early as 7–8 days after infection and peaked around 3 weeks (Figure 3A). Viral replication was paralleled by CD4^+^ T cell depletion in blood and tissues (Figure 3, B and C). Viral infection was detected by cell-associated HIV-1 RNA (cavRNA) quantification in various tissues of infected mice, showing clear viral dissemination (Supplemental Figure 6A). Levels of CD16 expression and functions of NK cells in blood, lymphoid and nonlymphoid tissue were enhanced during acute infection, albeit the enhancement in lymphoid tissues was less robust (Figure 3, D and E, and Supplemental Figure 6B). CD8^+^ T cells exhibited similar functional patterns with reduced degranulation and proinflammatory cytokine production in the LNs (Supplemental Figure 7). Previous studies showed that several immune checkpoint receptors (ICRs) were upregulated in NK cells in the setting of cancer or chronic viral infection and that these receptors negatively regulated NK cell cytotoxicity (47–50). Similarly, we found rapid increases of KLRG1, LAG-3, PD-1, and TIGIT expression in blood and tissue NK cells within 2 weeks after HIV-1 infection (Figure 3F and Supplemental Figure 6C). ICR upregulation in CD8^+^ T cells was also observed in the same groups of mice 21 days after HIV-1 infection (Supplemental Figure 8). Although NK cells were more functionally active during acute infection, upregulation of ICRs might lead to functional impairment. To further evaluate the possibility of functional impairment in NK cells during the course of HIV infection (Figure 4A), we monitored the survival, proliferation, and functionality of NK cells from day 0 to day 168 after infection. Rapid expansion of NK cells was seen in all organs (Figure 4B). A slow decline in NK numbers was seen between day 42 and 168. Correspondingly, NK cells proliferated in the organs early during the course of infection and returned to baseline when the infection was sustained beyond 100 days (Figure 4C). After ex vivo stimulation with PMA/ionomycin, NK cells at an early stage of infection showed increased potency for degranulation and IFN-γ and GZMB production, which then declined during the chronic phase (Figure 4, D–F). Notably, without ex vivo stimulation, the baseline functions of NK cells from acutely and chronically infected mice were similar and were higher than those from uninfected mice (Supplemental Figure 9), suggesting continuous NK cell activation. Taken together, these results suggest that HIV-1 results in chronic activation and functional impairment of NK cells in all tissues.

Liver NK cells had the highest percentage degranulation and cytokine production when compared with NK cells in other organs, but NK cells in all organs showed increased functionality after HIV infection that persisted throughout the studied span of disease. Additionally, the liver NK cells had the least decline in functionality throughout the course of infection when compared with the other organs. These data suggest that liver NK cells retained their functionality throughout the course of infection, possibly through the establishment of immunological memory in the liver (3, 51).

### ART prevents the progressive loss of NK cell functionality.

**Figure 5:**
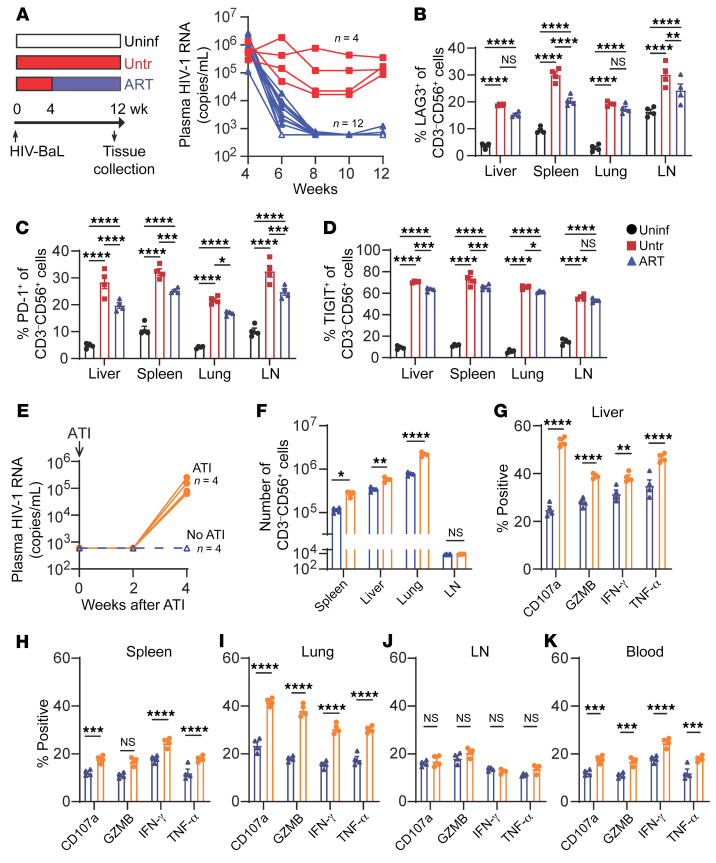
ART restores NK cell responses during HIV-1 infection. MISTRG-6-15 mice were infected with HIV-1_BAL_. (**A**) Infection and treatment scheme for the uninfected (white), untreated (red), and ART (blue) groups. Copies of plasma HIV-1 RNA were measured by RT-qPCR for the untreated and ART groups. (**B**–**D**) Percentage of tissue NK cells positive for KLRG1, LAG3, PD-1, and TIGIT. (**E**) viral load measurement after ATI. Lines connect data from the same mice. (**F**) Number of CD3^–^CD56^+^ NK cells in spleen, liver, lung, and LN 4 weeks after ATI. (**G**–**K**) Percentage of CD107a^+^, GZMB^+^, IFN-γ^+^, and TNF-α^+^ NK cells in indicated tissues. NK cells purified from tissues were stimulated with PMA/ionomycin ex vivo for 4 hours before flow cytometry analysis. Data displayed as mean ± SEM. In **B**–**K**, 4 mice were used for each time point. In **B**–**D** and **F**–**K**, *P* values were calculated using 2-way ANOVA with Šidák’s multiple comparison test. *P < 0.05, **P < 0.01, ***P < 0.001, and ****P < 0.0001.

In people living with HIV-1, functional impairment of NK cells due to chronic HIV-1 infection can be partially restored by ART (52–54). In the MISTRG6-15 mouse model, the expansion and functional activation of NK cells occurred within the first 10 days of infection, whereas NK cell numbers and functions began to decline on day 50 and almost returned to baseline after day 100 (Figure 4). Notably, the levels of inhibitory receptors including LAG3, PD-1, and TIGIT in NK cells increased during acute infection (Figure 3F), suggesting that early ART initiation may better improve NK cell functional restoration. To test this hypothesis, we studied mice that were initially infected for 4 weeks and then compared NK cells in mice on ART for 8 weeks to those from untreated mice (Figure 5A). The levels of inhibitory receptors including LAG-3, PD-1, and TIGIT all increased on NK cells after infection and were partially reduced in mice receiving ART (Figure 5, B–D). Next, we performed analytic treatment interruption (ATI) to determine whether NK cell functions were preserved by ART. Mice under ART had undetectable viral load, and virus rebounded in all animals within 4 weeks after ATI (Figure 5E). We found that the number of NK cells in various tissues increased following ATI (Figure 5F). In addition, NK cell functions including cytokine production and cytotoxicity were also increased after ATI (Figure 5, G–K). Notably, LN NK cells responded to initial infection (Figure 3) but did not respond to viral rebound after ATI, suggesting that additional approaches might be needed for the functional restoration of LN NK cells. Nonetheless, for nonlymphoid NK cells, these results suggest that ART prevented their functional impairment — including the loss of proliferation capacity and degranulation seen in chronically infected mice (Figure 4) — and ART reinvigorated NK cells in response to HIV-1 rebound after ATI.

### NK cells suppress HIV-1 infection in vivo.

**Figure 6:**
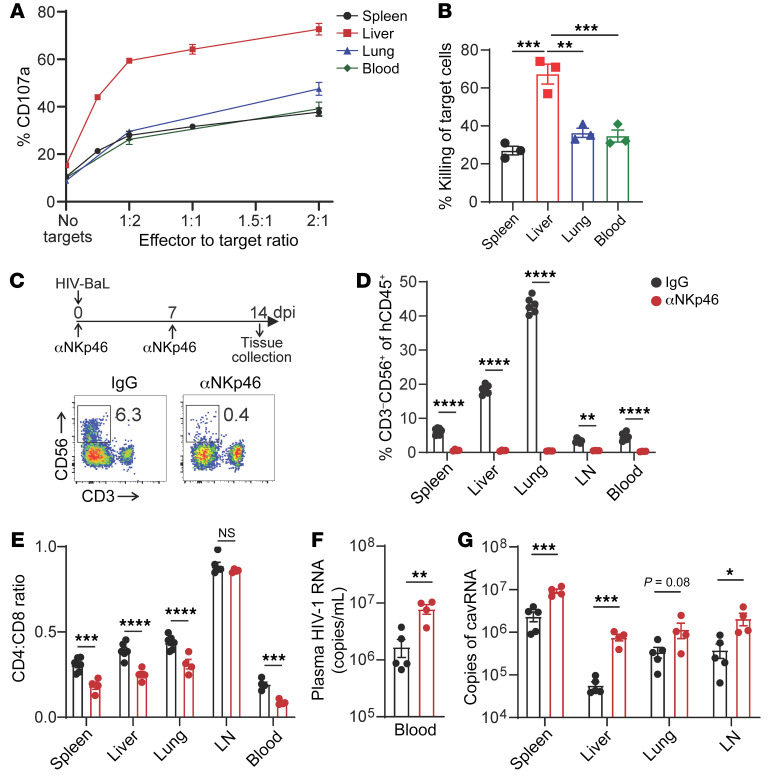
NK cells exhibit direct anti-HIV-1 control in vivo. MISTRG-6-15 mice were infected with HIV-1_BAL_. (**A** and **B**) NK cell degranulation and target cell killing. CD4^+^ T cells were purified from uninfected mice and then infected with HIV-1 reporter virus NL4-3-ΔEnv-EGFP. Autologous NK cells were purified from indicated tissues from infected mice and then cocultured with infected CD4^+^ T cells for 4 hours at indicated effector-to-target ratio. (**A**) NK cell degranulation and (**B**) live/dead staining of HIV-1–infected target cells (GFP^+^) was determined by flow cytometry. Cells were purified from 3 mice. (**C** and **D**) NK depletion by αNKp46 antibodies. Percentage of CD3^–^CD56^+^ NK cells in mouse tissues with or without αNKp46 antibody treatment. (**E**) CD4:CD8 ratio with or without αNKp46 antibody treatment. (**F** and **G**) Copies of plasma HIV-1 RNA (**F**) and copies of cavRNA in tissues (**G**) with or without αNKp46 antibody treatment. Data displayed as mean ± SEM. 5 mice were used in the isotype-treated group and 4 in the αNKp46-treated group. In (**B**) *P* values were calculated using 1-way ANOVA with Tukey’s multiple comparison post test. In (**D**, **E**, and **G**) *P* values were calculated using 2-way ANOVA with Šidák’s multiple comparison test. In **F**, *P* value was calculated using unpaired, 2-tailed *t* test. *P < 0.05, **P < 0.01, ***P < 0.001, and ****P < 0.0001.

Previous studies showed that transfusion of human blood NK cells suppressed HIV infection in humanized mice (55, 56). While our studies here show NK cells responded to HIV-1 infection as well as viral rebound after ATI, it is unclear whether endogenous NK cells can directly impact HIV-1 replication in vivo. To address this, we first isolated NK cells from various organs of infected mice and cocultured them with HIV-infected donor-matched CD4^+^ T cells for 4 hours. NK cells from all of the organs did exhibit degranulation and killed HIV-1–infected target cells, but liver NK cells showed the most pronounced response (Figure 6, A and B). Next, the in vivo control of HIV-1 by NK cells was studied by depleting NK cells with a monoclonal antibody against NKp46 that has been shown to deplete human NK cells in humanized mice (57). Here, depletion of NK cells was successful in all studied organs (Figure 6, C and D). On day 14 after HIV-1 infection CD4:CD8 ratios were significantly lower upon NK cell depletion compared with non-NK cell depleted mice, wit the exception of the LNs (Figure 6E). No apparent CD4 depletion was observed in LNs, even in the control mice, which is likely due to the small number of CD4^+^ T cells that express CCR5 (Supplemental Figure 10). Next, we collected blood and tissues to measure plasma HIV-1 RNA and cell-associated HIV-1 RNA, respectively. NK depletion led to an increase in plasma HIV-1 RNA by 5–10 fold (Figure 6F). Depletion of NK cells also caused increased cell-associated viral RNA in tissues, especially in the liver (Figure 6G), which was consistent with the robust NK cell response in the liver. These results strongly suggest that NK cells directly suppress HIV-1 replication in vivo.

### Antibody treatment improves NK cell functionality.

**Figure 7:**
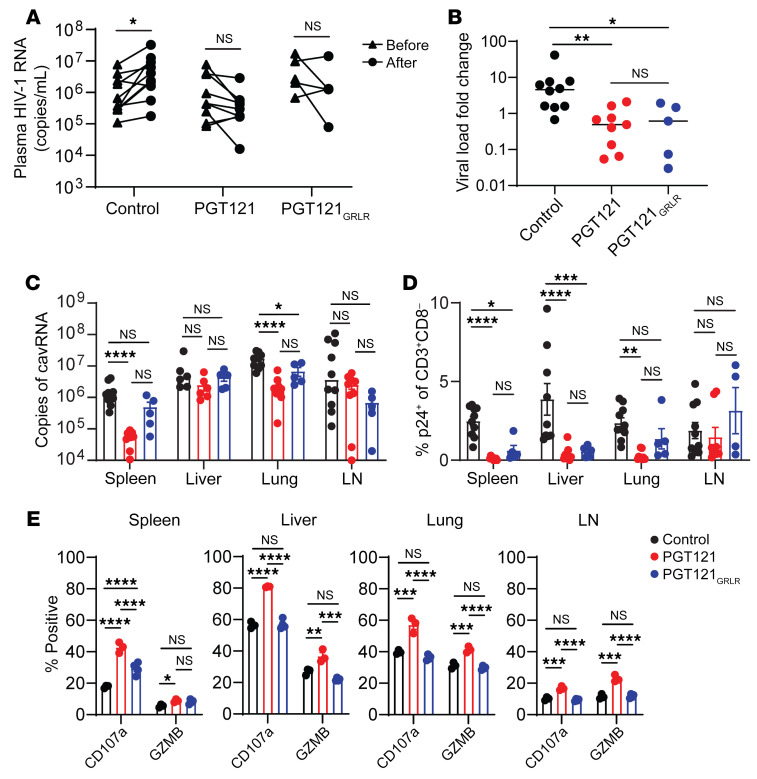
PGT121 antibody-mediated viral suppression enhances NK cell functionality. MISTRG-6-15 mice were infected with HIV-1_BAL_. 2 doses (20 mg/kg) of PGT121 (*n* = 9), PGT121_GRLR_ (*n* = 5), or PBS (*n* = 10) were administered on days 14 and 17 after infection. Blood samples were collected on days 14 and 20. Tissues were collected on day 20. (**A**) Plasma viral load before and after antibody treatment. Lines connect individual mice. *P* values were calculated using 2-way ANOVA with Šidák’s multiple comparison test. (**B**) Plasma viral load fold change from day 14 to day 20. *P* values were calculated using 1-way ANOVA with Tukey’s multiple comparison post test. (**C**) Copies of cavRNA in spleen, lung, and LN. (**D**) Percentage p24^+^ of CD3^+^CD8^–^ cells in spleen, lung, and LN. (**E**) Expression of CD107a or GZMB in NK cells in the spleen, lung, and LN. NK cells purified from tissues were stimulated with PMA/ionomycin ex vivo for 4 hours before flow cytometry analysis. PGT121 (*n* = 3), PGT121_GRLR_ (*n* = 4), and PBS (*n* = 3). Data displayed as mean ± SEM. In (**C**–**E**) *P* values for each tissue were calculated using 2-way ANOVA with Tukey’s multiple comparison post test. *P < 0.05, **P < 0.01, ***P < 0.001, and ****P < 0.0001.

Administration of broadly neutralizing antibodies (bNab) into viremic individuals enhances viral-specific T cell responses (58–62) and may also modulate NK cell functions through Fc-dependent mechanisms. To address the potential role that bNab therapy may have on NK responses, we used the HIV-neutralizing antibody PGT121 (63) to understand the Fc-dependent NK cell functions. Although IgM^+^ or IgD^+^ human B cells develop properly in most humanized mice, a scarce amount of hypermutated, class-switched IgG antibodies are produced, mainly due to the lack of germinal center response (64), necessitating a passive antibody treatment approach to study ADCC by NK cells. Moreover, we also used PGT12 with GRLR mutations that block antibody binding to Fc receptors (46). PGT121 or PGT121_GRLR_ was injected into infected mice, and viral loads in the plasma were reduced by both antibodies (Figure 7, A and B). In mice treated with PGT121, the spleen and lung had greater reduction of cavRNA and numbers of infected cells than the PGT121_GRLR_ group (Figure 7, C and D), indicating Fc-dependent clearance of HIV-1–infected cells. No reduction was seen in the LNs, likely due to the lack of functionally mature NK cells in the LNs. Surprisingly, the number of HIV-p24^+^ cells in the liver was reduced by PGT121, whereas liver HIV-1 RNA was unchanged. It is possible that HIV-1 RNA signals were detected from kupffer cells that engulfed HIV-1–infected T cells upon antibody treatment. Since the GRLR mutation abolishes both ADCC (Supplemental Figure 4) and ADCP (46), it is possible that macrophages and NK cells both contributed to the clearance of HIV-1–infected cells mediated by PGT121 interacting with the intact Fc fragment. Next, we aimed to evaluate the influence of antibody therapy on NK cells. NK cell functions such as degranulation and GzmB production were selectively enhanced by PGT121 in the spleen, liver, and lung, and were slightly enhanced in the lymph nodes. By contrast, the PGT121_GRLR_ did not have any effect on NK cell functionality (Figure 7E). These results support the hypothesis that antibody therapies improve NK cell cytolytic activity in an Fc-dependent manner.

## Discussion

Despite strong epidemiological evidence from large-scale cross-sectional studies, there is still a nascent understanding of the roles that NK cells play in HIV-1 infection in humans. The lack of clarity is likely due to several factors: the dependence of NK cell function on the associated HLA genotype, the functional diversity not fully captured by examination of circulating NK cells, and finally, the inadequacy of animal models. The MISTRG-6-15 mouse model recapitulates the human immune system beyond earlier humanized mouse models. Of note, the NK population is robust and more functional than it is in NSG mice, and it better mirrors human NK responses to immunological challenges in the various organs. More importantly, the various organs also showed dramatically different responses and functionality of the NK cells, especially when comparing lymphoid and nonlymphoid tissues. While NK cells from lymphoid tissues from human donors had reduced functionality when compared with blood, human NK cells in MISTRG-6-15 mice had similar properties. Thus, human NK cells in reconstituted MISTRG-6-15 mice more closely resembled NK cells in humans than prior humanized mouse models.

Here, we further showed that, in humanized MISTRG-6-15 mice infected with HIV-1, the depletion of NK cells by monoclonal antibody resulted in higher levels of viral replication and accelerated loss of CD4^+^ T cells. For the first time, we found the direct evidence for control of HIV-1 infection by NK cells in vivo. Furthermore, we found no change in disease progression in the LNs after NK depletion, consistent with the reduced functionality of human NK cells in lymphoid tissue. Thus, this mouse model allows us to study NK responses to HIV-1, especially in different organs and lymphoid versus nonlymphoid tissues.

The humanized MISTRG-6-15 mice also allowed investigation of how bNab therapy modulates NK cell functions, as related to antibody effector functions. In HIV-1–infected mice, NK cell functionality was improved with PGT121 administration in all organs, but was least improved in the lymph nodes. These results highlight the potential difficulty in eliminating the remaining infected cells in lymphoid tissues by bNabs, perhaps due to the lack of functional effector cells.

During acute HIV-1 infection in humanized MISTRG-6-15 mice, NK cells rapidly expanded, and greater percentages of cells degranulated and produced inflammatory cytokines in nonlymphoid tissues, while those in lymphoid tissues were immature or dysfunctional throughout the course of infection. This was consistent with results in humans and studies of nonhuman primates infected with SIV (65). This highlights a key concern of HIV-1 persistence in lymphoid tissue (34, 66, 67), as overall, the NK cells appear to be less cytotoxic and proinflammatory. In chronically infected mice, the functions of NK cells declined in various tested organs, which was accompanied by an increase in potential NK exhaustion markers in all of the organs studied, which may allude to overall dampened response to HIV-1 by these cells over time. The dampened response was partially reduced after ART, which resulted in the return of functional NK cells after treatment interruption. It is notable that NK cells in the LNs were not strongly affected by ART; this suggests that either (a) overall, NK cells minimally responded to HIV-1 in the lymphoid tissue initially or (b) immunological stimulation was continuous due to the persistence of HIV-1–infected cells in the lymphoid tissues despite ART (68).

By better understanding the role of NK cells during the course of HIV-1 infection in different organs, more targeted therapies and approaches to treatment can be pursued. Therefore, going forward, it will be important to use this humanized mouse model to dissect the molecular and cellular mechanism for NK cell-mediated HIV-1 suppression and to develop new strategies to enhance NK cell functions, especially in the lymphoid tissues.

## Methods

### Mouse strains.

The generation of knock-in mice encoding human M-CSF, IL3/GM-CSF, *SIRPA*, THPO, IL6, and *IL15* in a 129xBALB/c (N3) genetic background (42, 44) was performed using Velocigene technology (Regeneron Pharmaceuticals). Mice were bred to a *Rag2^−/−^ Il2rg^−/−^* background with homozygous human gene knock in (^h/h^) to generate 2 mouse colonies, including MCSF^h/h^ GMCSF^h/h^ IL3^h/h^ THPO^h/h^ IL6^h/h^ *Rag2^−/−^ Il2rg^null^* and MCSF^h/h^ GMCSF^h/h^ IL3^h/h^ *SIRPA*^h/h^ THPO^h/h^ IL6^h/h^ *IL15*^h/h^ *Rag2^−/−^ Il2rg^null^*. To produce mice for engraftment with human cord blood CD34^+^ cells, the 2 colonies were crossed to generate MCSF^h/h^ IL3^h/h^GMCSF^h/h^ *SIRPA*^h/m^ THPO^h/h^ IL6^h/h^ *IL15*^h/m^ *Rag2^−/−^ Il2rg^−/−^* mice (where ^h/m^ indicates human/mouse heterozygous gene knockin), labeled MISTRG-6-15. Human SIRPA and IL15 loci were used as heterozygotes for engraftment throughout the study. Nonobese diabetic SCID *Il2rg*^null^ (NSG) mice were obtained from The Jackson Laboratory.

### Human samples.

Deidentified human cord blood samples were collected at the St. Louis Cord blood bank. Anonymous peripheral blood samples were acquired from the Mississippi Valley Regional Blood Center as waste cellular products. Human tonsils were collected from elective tonsillectomies from Children’s Hospital in Saint Louis, which were provided as surgical waste, with no identifiers attached.

### Plasmids, monoclonal antibodies, and viruses.

The anti-HA and anti-HA-GRLR IgG1 heavy chain- and light chain- expressing plasmids as well as the HIVivo-HA viral plasmid were obtained from Michel Nussenzweig laboratory at The Rockefeller University (New York, New York, USA) (46). Codon optimized PGT121 heavy chain and light chain expressing plasmids were obtained from Dennis Burton laboratory at The Scripps Research Institute (La Jolla, California, USA). GRLR mutations were introduced to the PGT121 heavy chain plasmid. PGT121 and anti-HA antibodies were produced by transfecting the FreeStyle 293-F cells. Replication-competent HIV_BaL_ viruses were prepared from PHA-stimulated, CD8-depleted, healthy-donor PBMCs. The HIV-1 reporter virus NL4-3-ΔEnv-EGFP (AIDs reagent program) pseudotyped with VSVG envelope was prepared from transient transfection of plasmid DNA into 293T cells. HIVivo-HA virus was also prepared from transient transfection of 293T cells. Concentrated viral stocks were prepared using Lenti-X Concentrator (TaKaRa).

### In vitro ADCC assay.

Deidentified frozen tonsillar and blood mononuclear cells were used for the functional analyses. To obtain purified human NK cells from blood and tonsil mononuclear cells, CD3/CD19 depletion was performed before NK cell purification using EasySep Human NK Cell Isolation Kit (Stemcell Technologies). Human CD4^+^ T cells were isolated using MojoSort Human CD4 T Cell Isolation Kit (Biolegend). Purified CD4^+^ T cells were costimulated with plate-bound CD3 (Biolegend, 300465) and soluble CD28 (Biolegend, 302943) antibodies with the presence of 20 ng/mL IL-2 (Biolegend, 589106) for 3 days prior to viral infection. Activated CD4^+^ cells were infected with HIVivo-HA virus by spin inoculation at 1,200*g* for 2 hours at 30°C and incubated at 37°C in RPMI1640 medium (GIBCO) containing 10% FBS supplemented with 20 ng/mL IL-2. Four days after infection, infected tonsillar or blood CD4^+^ T cells were cocultured with their autologous NK cells in a 1:1 effector:target ratio with anti-HA or anti-HA_GRLR_ (1 μg/mL) antibody for 4 hours. Target cell lysis was determined by the percentage of lysed infected cells (CD3^+^CD8^–^HA^+^7-AAD^+^) in the total infected cells (CD3^+^CD8^–^HA^+^).

### Generation of humanized mice.

Human CD34^+^ cells were isolated from cord blood using EasySep Human Cord Blood CD34 Positive Selection Kit II (Stemcell Technologies) and were cryopreserved in IMDM containing 7.5% DMSO. For MISTRG-6-15 mice, 1–3 day-old newborn mice were humanized through injection of 1.5–3×10^4^ cord blood CD34^+^ cells intrahepatically. For NSG mice, 1–3 day-old newborn mice preconditioned with sublethal irradiation (80 cGy) followed by intrahepatic injection of 1×10^5^ cord blood CD34^+^ cells. Reconstitution of human CD45^+^ cells in blood was determined 9–10 weeks after engraftment. Mice were grouped after checking for blood engraftment to ensure that animals from different treatment groups or time points had similar levels of human T cells, NK cells, and macrophages. In each treatment group and at any time point, both male and female mice were used. Mice were randomly sorted into different treatment groups and time points. Identical cord blood donors were used when possible for experiments, but variation in donors between experiments does exist.

### HIV-1 infection and treatment of humanized mice.

Nine to 10 weeks after engraftment, the MISTRG-6-15 mice were infected with HIV-1_BAL_ (10 ng p24) by retroorbital injection. Uninfected mice received 100 μl PBS by retroorbital injection. To quantify HIV-1 infection in tissues by flow cytometry, intracellular HIV-p24 staining (Beckman Coulter) was performed using the Cytofix/Cytoperm kit (BD Biosciences). To quantify plasma HIV-1 RNA, blood samples were collected by retroorbital or submandibular bleeding. Plasma viral RNA was extracted by Quick-RNA Viral Kits (Zymo Research) before reverse transcription using SuperScript III Reverse Transcriptase (Thermo Fisher Scientific). Quantification of tissue HIV-1 RNA was described previously (69). Briefly, after single cell suspensions of tissues were obtained as described below, a fixed portion of each tissue was used for RNA extraction by Direct-zol RNA Kits (Zymo Research), then reverse transcribed into cDNA using SuperScript III reverse transcriptase (Invitrogen). The HIV-1 *gag*-based qPCR assays using 10-fold serial dilutions of HIV-1 genomic DNA as standard (70) were used to quantify plasma- and tissue-HIV-1 RNA. Total HIV-1 copy numbers were then obtained by multiplying by the proportion of the total sample used for analysis.

To suppress HIV-1 replication in mice, antiretrovirals were added into mouse food as previously described (71). Briefly, final concentrations of drugs in mouse food are 1 g/kg tenofovir disoproxil fumarate, 1 g/kg emtricitabine, and 2 g/kg raltegravir. To deplete human NK cells in vivo, mouse anti-human NKp46 antibodies (Cell Sciences, clone B-L46) were administered by i.p. injection on day 0 and day 7 post infection. Each mouse received 50 μg antibody per injection. Control mice received 100 μL PBS by i.p. injection. For bNab treatment, PGT121 or PGT121_GRLR_ was administered by retroorbital injection. Each mouse received 250 μg antibody per injection. Control mice received 100 μl PBS by retroorbital injection.

### Functional analysis of NK cells and CD8^+^ T cells from humanized mice.

Spleens, livers, and submandibular, axillary, and thoracic LNs were excised from mice and filtered through 100 μm strainers using the back end of a syringe. The strainer was washed with tissue buffer made of PBS with FBS. Samples were centrifuged followed by resuspension of the pellet in tissue buffer. For livers, cells were resuspended in 8 mL of tissue buffer and 5 mL of 100% percoll and then centrifuged for 20 minutes at 760*g*. Lungs were excised and cut into small pieces with scissors. They were then incubated at 37°C with digestion buffer contained collagenase and DNase I for 45 minutes. Samples were then filtered through 100 μm strainers using the back end of a syringe. The strainer was washed with tissue buffer. Samples were centrifuged followed by resuspension of pellet in 10 mL of 40% percoll. Samples were centrifuged for 20 minutes at 760*g*. After single-cell suspensions from all tissues were obtained, cells were utilized for 3 purposes, including ICR staining, intracellular cytokine staining, and target cell lysis. ICR staining was performed using freshly isolated cells. For intracellular cytokine production, cells were stimulated with PMA (50 ng/mL) and ionomycin (500 ng/mL) and stained with anti-CD107a antibody for 4 hours. Brefeldin A and monensin (golgiplug) were used for the final hour of stimulation. Cells were then washed and stained with CD56, CD3, CD8, NKp44, and CD16. Cytofix/Cytoperm kit (BD Biosciences) was then utilized and intracellular staining was performed with IFN-γ, TNF-α and GZMB (Supplemental Figure 11). Purified NK cells were used to assess target cell lysis. To purify NK cells from mice, single cell suspensions were obtained from specified organs as outlined above followed by elimination of CD45^+^ mouse cells with EasySep Mouse CD45 Positive Selection Kit (Stemcell Technologies) followed by NK cell isolation using EasySep Human NK Cell Isolation Kit (Stemcell Technologies). Purified NK cells were coincubated with CFSE-labeled (Thermo Fisher Scientific) K562 cells (ATCC), with effector-to-target ratios of 1:2, for 4 hours, and cytotoxicity was assayed by 7-AAD uptake. Background spontaneous K562 blast death (measured via the no effector control wells) was subtracted from the total to yield the percentage of K562 cell–specific cytotoxicity. For experiments investigating perforin-mediated killing, concanamycin A (Sigma-Aldrich) was utilized at 100 nM with cells incubated for 120 minutes prior to the killing assay. To determine lysis of HIV-1–infected target cells, NK cells and CD4^+^ T cells were purified separately from mice engrafted with CD34^+^ cells from the same cord blood donor. Purified CD4^+^ T cells were infected with the HIV-1 reporter virus NL4-3-ΔEnv-EGFP for 3 days. Infected cells were then cocultured with freshly isolated NK cells from various tissues in a 1:1 effector-to-target ratio for 4 hours and cytotoxicity was assayed by 7-AAD uptake in GFP^+^ cells.

### Flow cytometry analysis.

Flow cytometry was performed using BD LSRFortessa or BD Canto II and data were analyzed by Flowjo software. Antibodies used for surface and intracellular staining were purchased from Biolegend and include mCD45 (clone 30-F11), hCD45 (clone HI30), hCD3 (clone HIT3a), hCD4 (clone OKT4), hCD8 (clones HIT8a and RPA-T8), hCD14 (clone M5E2), hNKp46 (clone 9E2), hCD56 (clone HCD56), hCD16 (clone 3G8). Antibodies purchased from eBioscience include hCD3 (clone UCHT1), hCD16 (clone CB16), from Beckman Coulter include anti-HIV-1 p24 (clone KC57-RD1), and from NIH AIDS Reagent Program include anti-HIV-1 p24 (clone KC57). Additional antibodies were purchased from BD Biosciences and include hCD56 (clone NCAM16.2), hCD3 (clone SK7), hCD107a (clone H4A3), hIFN-γ (clone 4S.B3), hTNF-α (clone mAB11), and 7AAD (51-2359KC) from BD Pharmingen, and hGranzymeB (clone GB12) from Invitrogen.

### Statistics.

Data shown are mean values with error bars denoting SEM. In figures comparing 2 groups, *P* values were calculated using unpaired, 2-tailed *t* tests. When more than 2 groups were compared, 1-way ANOVA was utilized with Tukey’s multiple comparison post test. In figures comparing multiple groups with multiple outcomes or organs, *P* values were calculated using 1-way ANOVA with Tukey’s multiple comparison post test, or 2-way ANOVA with Šidák’s multiple comparison test or Tukey’s multiple comparison post test. Analysis was performed with GraphPad Prism 8 (Graphpad Software). A *P* value less than 0.05 was consider significant.

### Study approval.

All animal experiments were approved by the Institutional Animal Care and Use Committee of Washington University School of Medicine, approval no. 20-0224. Human cord blood and tonsil samples were classified as surgical waste with no identifiers attached and did not require further approval for usage.

## Author contributions

CMS, WMY, and LS designed the study and wrote the manuscript; CMS, QW, ANO, and LS performed in vitro and animal experiments and analyzed the data; HG and AJS performed antibody production; MC contributed to the analysis of NK cell functions in tonsils.

## Supplementary Material

Supplemental data

## Version 1 10/25/2022

In-Press Preview

## Version 2 12/15/2022

Electronic publication

## References

[1] Richard J (2010). HIV-1 Vpr up-regulates expression of ligands for the activating NKG2D receptor and promotes NK cell-mediated killing. Blood.

[2] Ward J (2007). HIV modulates the expression of ligands important in triggering natural killer cell cytotoxic responses on infected primary T-cell blasts. Blood.

[3] Nikzad R (2019). Human natural killer cells mediate adaptive immunity to viral antigens. Sci Immunol.

[4] [No authors listed] (1990). The FC and not CD4 receptor mediates antibody enhancement of HIV infection in human cells. Dis Markers.

[5] Ahmad A (1994). Evidence for a defect of antibody-dependent cellular cytotoxic (ADCC) effector function and anti-HIV gp120/41-specific ADCC-mediating antibody titres in HIV-infected individuals. J Acquir Immune Defic Syndr (1988).

[6] Apps R (2016). HIV-1 Vpu mediates HLA-C downregulation. Cell Host Microbe.

[7] Cohen GB (1999). The selective downregulation of class I major histocompatibility complex proteins by HIV-1 protects HIV-infected cells from NK cells. Immunity.

[8] Martin MP (2002). Epistatic interaction between KIR3DS1 and HLA-B delays the progression to AIDS. Nat Genet.

[9] Martin MP (2007). Innate partnership of HLA-B and KIR3DL1 subtypes against HIV-1. Nat Genet.

[10] Boulet S (2008). A combined genotype of KIR3DL1 high expressing alleles and HLA-B*57 is associated with a reduced risk of HIV infection. AIDS.

[11] Boulet S (2008). Increased proportion of KIR3DS1 homozygotes in HIV-exposed uninfected individuals. AIDS.

[12] Luo M (2018). KIR3DL1 alleles and their epistatic interactions with human leukocyte antigen class I influence resistance and susceptibility to HIV-1 acquisition in the Pumwani sex worker cohort. AIDS.

[13] Alter G (2011). HIV-1 adaptation to NK-cell-mediated immune pressure. Nature.

[14] Ackerman ME (2016). Polyfunctional HIV-specific antibody responses are associated with spontaneous HIV control. PLoS Pathog.

[15] Baum LL (1996). HIV-1 gp120-specific antibody-dependent cell-mediated cytotoxicity correlates with rate of disease progression. J Immunol.

[16] Lambotte O (2009). Heterogeneous neutralizing antibody and antibody-dependent cell cytotoxicity responses in HIV-1 elite controllers. AIDS.

[17] Madhavi V (2017). HIV-1 Env- and Vpu-specific antibody-dependent cellular cytotoxicity responses associated with elite control of HIV. J Virol.

[18] Chung AW (2014). Polyfunctional Fc-effector profiles mediated by IgG subclass selection distinguish RV144 and VAX003 vaccines. Sci Transl Med.

[19] Chung AW (2015). Dissecting polyclonal vaccine-induced humoral immunity against HIV using systems serology. Cell.

[20] Haynes BF (2012). Immune-correlates analysis of an HIV-1 vaccine efficacy trial. N Engl J Med.

[21] Alter G (2007). Differential natural killer cell-mediated inhibition of HIV-1 replication based on distinct KIR/HLA subtypes. J Exp Med.

[22] Alter G (2009). HLA class I subtype-dependent expansion of KIR3DS1+ and KIR3DL1+ NK cells during acute human immunodeficiency virus type 1 infection. J Virol.

[23] Boudreau JE (2016). KIR3DL1 and HLA-B density and binding calibrate NK education and response to HIV. J Immunol.

[24] Boulet S (2010). HIV protective KIR3DL1 and HLA-B genotypes influence NK cell function following stimulation with HLA-devoid cells. J Immunol.

[25] Song R (2014). HIV protective KIR3DL1/S1-HLA-B genotypes influence NK cell-mediated inhibition of HIV replication in autologous CD4 targets. PLoS Pathog.

[26] Bjorkstrom NK (2022). Natural killer cells in antiviral immunity. Nat Rev Immunol.

[27] Sojka DK (2014). Tissue-resident natural killer cells and their potential diversity. Semin Immunol.

[28] Dogra P (2020). Tissue determinants of human NK cell development, function, and residence. Cell.

[29] Fehniger TA (2003). CD56bright natural killer cells are present in human lymph nodes and are activated by T cell-derived IL-2: a potential new link between adaptive and innate immunity. Blood.

[30] Freud AG (2006). Evidence for discrete stages of human natural killer cell differentiation in vivo. J Exp Med.

[31] Folkvord JM (2005). Lymphoid follicles are sites of heightened human immunodeficiency virus type 1 (HIV-1) replication and reduced antiretroviral effector mechanisms. AIDS Res Hum Retroviruses.

[32] Haase AT (1996). Quantitative image analysis of HIV-1 infection in lymphoid tissue. Science.

[33] Pantaleo G (1991). Lymphoid organs function as major reservoirs for human immunodeficiency virus. Proc Natl Acad Sci U S A.

[34] Perreau M (2013). Follicular helper T cells serve as the major CD4 T cell compartment for HIV-1 infection, replication, and production. J Exp Med.

[35] Victor Garcia J (2016). Humanized mice for HIV and AIDS research. Curr Opin Virol.

[36] Andre MC (2010). Long-term human CD34+ stem cell-engrafted nonobese diabetic/SCID/IL-2R gamma(null) mice show impaired CD8+ T cell maintenance and a functional arrest of immature NK cells. J Immunol.

[37] Strowig T (2010). Human NK cells of mice with reconstituted human immune system components require preactivation to acquire functional competence. Blood.

[38] Chen Q (2009). Expression of human cytokines dramatically improves reconstitution of specific human-blood lineage cells in humanized mice. Proc Natl Acad Sci U S A.

[39] Huntington ND (2009). IL-15 trans-presentation promotes human NK cell development and differentiation in vivo. J Exp Med.

[40] Cui G (2014). Characterization of the IL-15 niche in primary and secondary lymphoid organs in vivo. Proc Natl Acad Sci U S A.

[41] Castillo EF, Schluns KS (2012). Regulating the immune system via IL-15 transpresentation. Cytokine.

[42] Rongvaux A (2014). Development and function of human innate immune cells in a humanized mouse model. Nat Biotechnol.

[43] Ivic S (2017). Differential dynamics of HIV infection in humanized MISTRG versus MITRG mice. Immunohorizons.

[44] Herndler-Brandstetter D (2017). Humanized mouse model supports development, function, and tissue residency of human natural killer cells. Proc Natl Acad Sci U S A.

[45] Schurch CM (2014). Cytotoxic CD8+ T cells stimulate hematopoietic progenitors by promoting cytokine release from bone marrow mesenchymal stromal cells. Cell Stem Cell.

[46] Horwitz JA (2017). Non-neutralizing antibodies alter the course of HIV-1 infection in vivo. Cell.

[47] Anderson AC (2016). Lag-3, Tim-3, and TIGIT: co-inhibitory receptors with specialized functions in immune regulation. Immunity.

[48] Ito M (2006). Killer cell lectin-like receptor G1 binds 3 members of the classical cadherin family to inhibit NK cell cytotoxicity. J Exp Med.

[49] Ndhlovu LC (2012). Tim-3 marks human natural killer cell maturation and suppresses cell-mediated cytotoxicity. Blood.

[50] Stanietsky N (2009). The interaction of TIGIT with PVR and PVRL2 inhibits human NK cell cytotoxicity. Proc Natl Acad Sci U S A.

[51] Paust S (2010). Critical role for the chemokine receptor CXCR6 in NK cell-mediated antigen-specific memory of haptens and viruses. Nat Immunol.

[52] Chehimi J (2007). Baseline viral load and immune activation determine the extent of reconstitution of innate immune effectors in HIV-1–infected subjects undergoing antiretroviral treatment. J Immunol.

[53] Jensen SS (2015). HIV-specific antibody-dependent cellular cytotoxicity (ADCC) -mediating antibodies decline while NK cell function increases during antiretroviral therapy (ART). PLoS 1.

[54] Lichtfuss GF (2012). Virologically suppressed HIV patients show activation of NK cells and persistent innate immune activation. J Immunol.

[55] Kim JT (2022). Latency reversal plus natural killer cells diminish HIV reservoir in vivo. Nat Commun.

[56] Ni Z (2014). Expression of chimeric receptor CD4ζ by natural killer cells derived from human pluripotent stem cells improves in vitro activity but does not enhance suppression of HIV infection in vivo. Stem Cells.

[57] Chijioke O (2013). Human natural killer cells prevent infectious mononucleosis features by targeting lytic Epstein-Barr virus infection. Cell Rep.

[58] Borducchi EN (2018). Antibody and TLR7 agonist delay viral rebound in SHIV-infected monkeys. Nature.

[59] Caskey M (2019). Broadly neutralizing anti-HIV-1 monoclonal antibodies in the clinic. Nat Med.

[60] Niessl J (2020). Combination anti-HIV-1 antibody therapy is associated with increased virus-specific T cell immunity. Nat Med.

[61] Nishimura Y (2017). Early antibody therapy can induce long-lasting immunity to SHIV. Nature.

[62] Scheid JF (2016). HIV-1 antibody 3BNC117 suppresses viral rebound in humans during treatment interruption. Nature.

[63] Walker LM (2011). Broad neutralization coverage of HIV by multiple highly potent antibodies. Nature.

[64] Karpel ME (2015). BLT humanized mice as a small animal model of HIV infection. Curr Opin Virol.

[65] Florez-Alvarez L (2018). NK Cells in HIV-1 infection: from basic science to vaccine strategies. Front Immunol.

[66] Estes JD (2017). Defining total-body AIDS-virus burden with implications for curative strategies. Nat Med.

[67] Rabezanahary H (2020). Despite early antiretroviral therapy effector memory and follicular helper CD4 T cells are major reservoirs in visceral lymphoid tissues of SIV-infected macaques. Mucosal Immunol.

[68] Banga R (2016). PD-1(+) and follicular helper T cells are responsible for persistent HIV-1 transcription in treated aviremic individuals. Nat Med.

[69] Gao H Evaluation of HIV-1 latency reversal and antibody-dependent viral clearance by quantification of singly spliced HIV-1 *vpu*/*env* mRNA. J Virol.

[70] Deng K (2015). Broad CTL response is required to clear latent HIV-1 due to dominance of escape mutations. Nature.

[71] Halper-Stromberg A (2014). Broadly neutralizing antibodies and viral inducers decrease rebound from HIV-1 latent reservoirs in humanized mice. Cell.

