# Supplementary material for: Human NK cells confer protection against HIV-1 infection in humanized mice
Source: J Clin Invest. 2022 Dec 15;132(24):e162694. doi: 10.1172/JCI162694 (PMC9753998; doi:10.1172/JCI162694)
Supplement: Supplemental data [file jci-132-162694-s130.pdf]

Figure S1

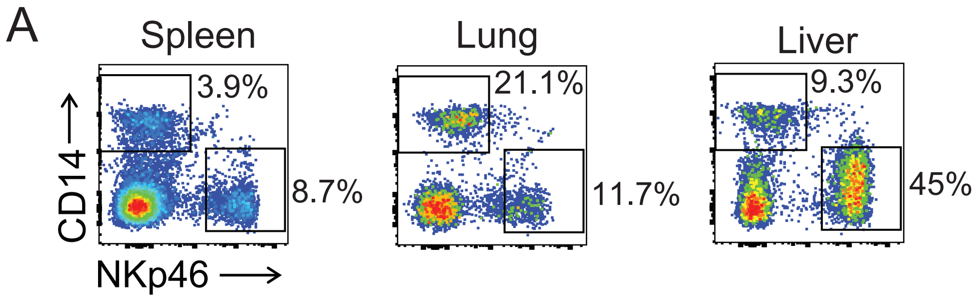
A
Spleen
Lung
Liver
3.9%
8.7%
21.1%
11.7%
9.3%
45%
CD14
NKp46

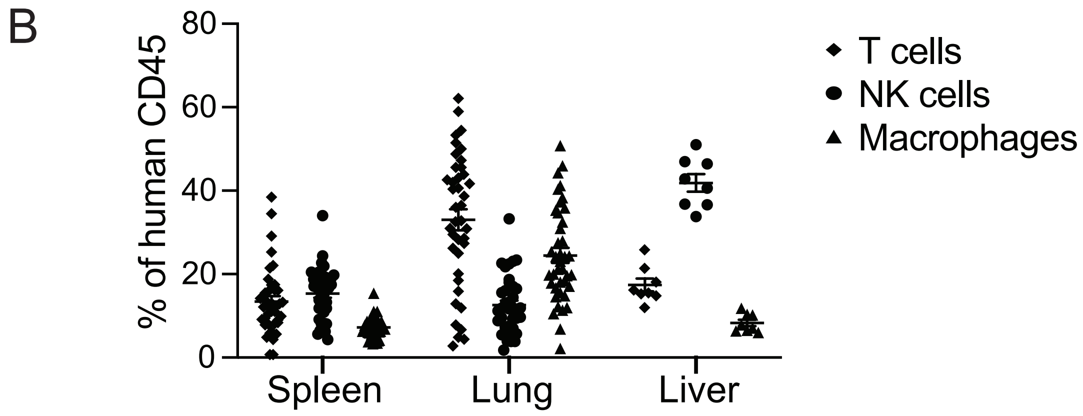
B
80
60
40
20
0
% of human CD45
Spleen
Lung
Liver
T cells
NK cells
Macrophages

Supplemental Fig. 1. Immune cell distribution in MISTRG-6-15 mice.

A, Representative flow of CD14 and NKp46 staining in spleen, lung and liver. **B,** Percentage of human T cells (CD3), NK cells (NKp46), and macrophages (CD14) in spleen, lung, and liver. Results from several independent cohorts were combined.

Figure S2

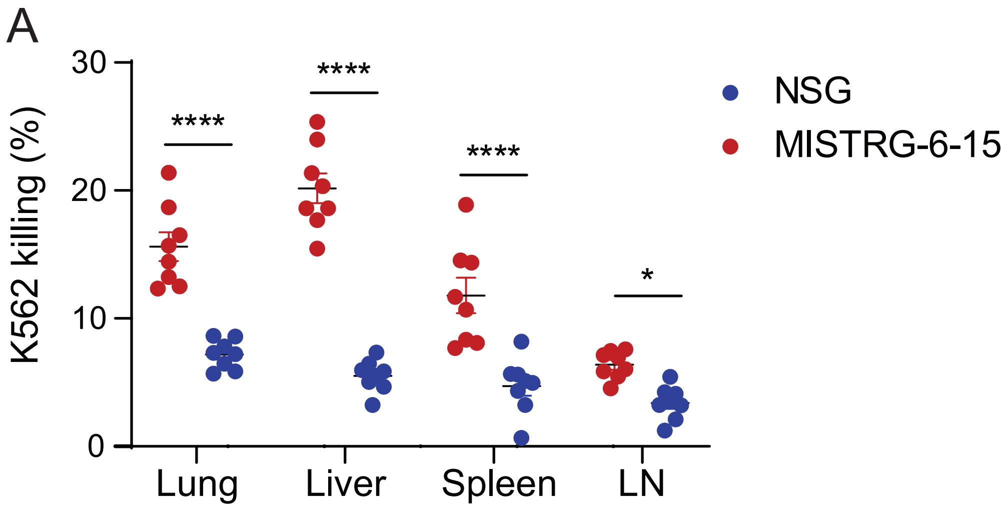
A
K562 killing (%)
30
20
10
0
****
****
****
*
Lung
Liver
Spleen
LN
NSG
MISTRG-6-15

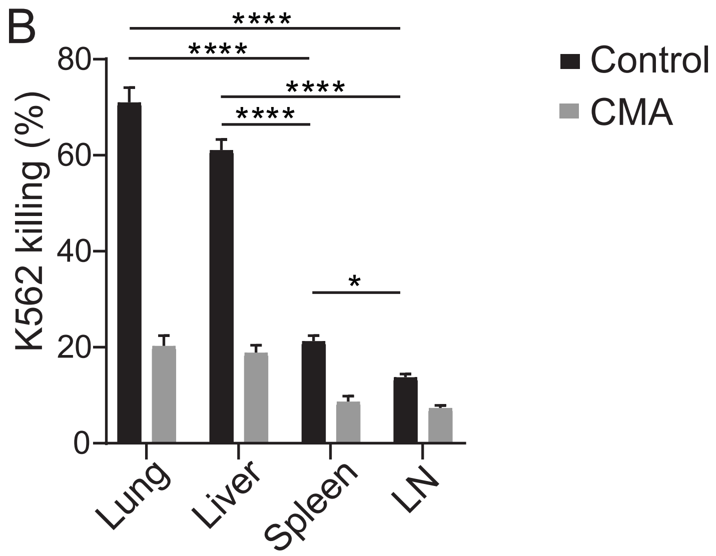
B
K562 killing (%)
80
60
40
20
0
****
****
****
****
*
Lung
Liver
Spleen
LN
Control
CMA

Supplemental Fig. 2. K562 killing by NK cells from NSG and MISTRG-6-15 mice.

A, ex vivo killing of K562 by purified tissue NK cells after 4 hours of co-culture. **B**, ex vivo killing of K562 by purified tissue NK cells from MISTRG-6-15 mice with and without concanamycin A. NK cells and K562 cells were co-cultured at 1:1 for 4 hours before flow cytometry analysis. Error bars show mean values with SEM. P-values were calculated using a two-way ANOVA with Tukey multi-comparison post-test. * denotes p-value <0.05, ** denotes p-value <0.01, *** denotes p-value <0.001, and **** denotes p-value <0.0001.

Figure S3

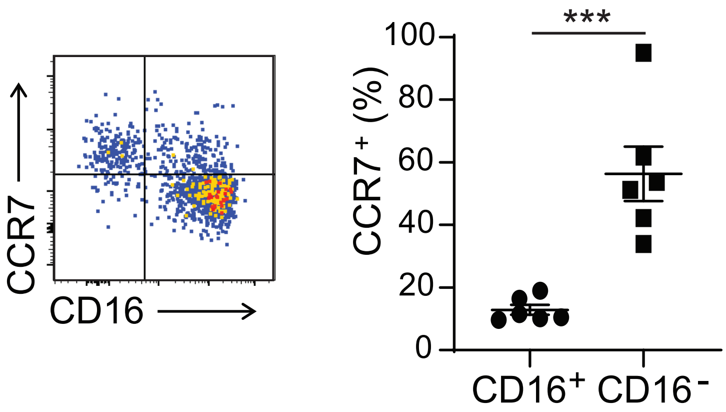

Supplemental Fig. 3. CCR7 and CD16 expression in NK cells.

Representative flow plot of CCR7 and CD16 expression in blood NK cells. Percentage of $CCR7^+$ in $CD16^+$ and $CD16^-$ NK cells. Error bars show mean values with SEM. Unpaired, two-tailed t-test used. *** denotes p-value <0.001.

Figure S4

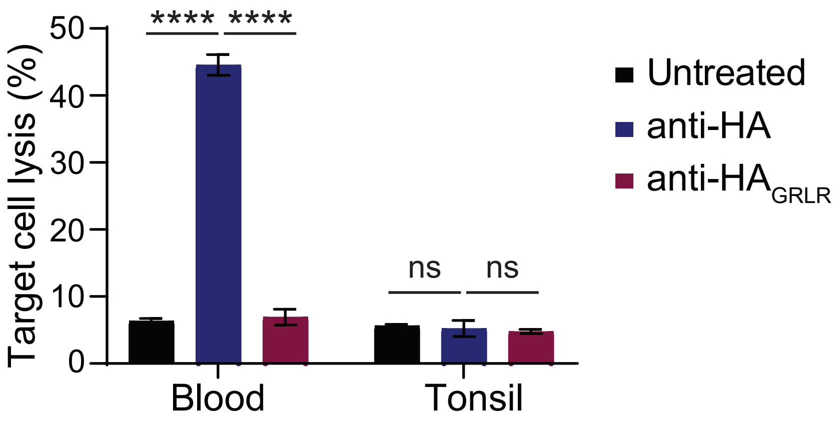

Target cell lysis (%)
50
40
30
20
10
0
****
****
ns
ns
Blood
Tonsil
Untreated
anti-HA
anti-HA$_{GRLR}$

Supplemental Fig. 4. Killing of HIV-1-infected $CD4^+$ cells by NK cells from blood and tonsil tissues.

$CD4^+$ T cells infected with HIVivo-HA virus were co-cultured with autologous NK cells from either blood or tonsils as noted at 1:1 for 4 hours with the presence of anti-HA or anti-HA_{GRLR} (1µg/ml). Target cells lysis was determined by the percentage of lysed infected cells ($CD3^+CD8^-HA^+$7-AAD^+) in the total infected cells ($CD3^+CD8^-HA^+$). Error bars show mean values with SEM. *P*-values were calculated using a two-way ANOVA with Tukey multi-comparison post-test. **** denotes p-value <0.0001.

Figure S5

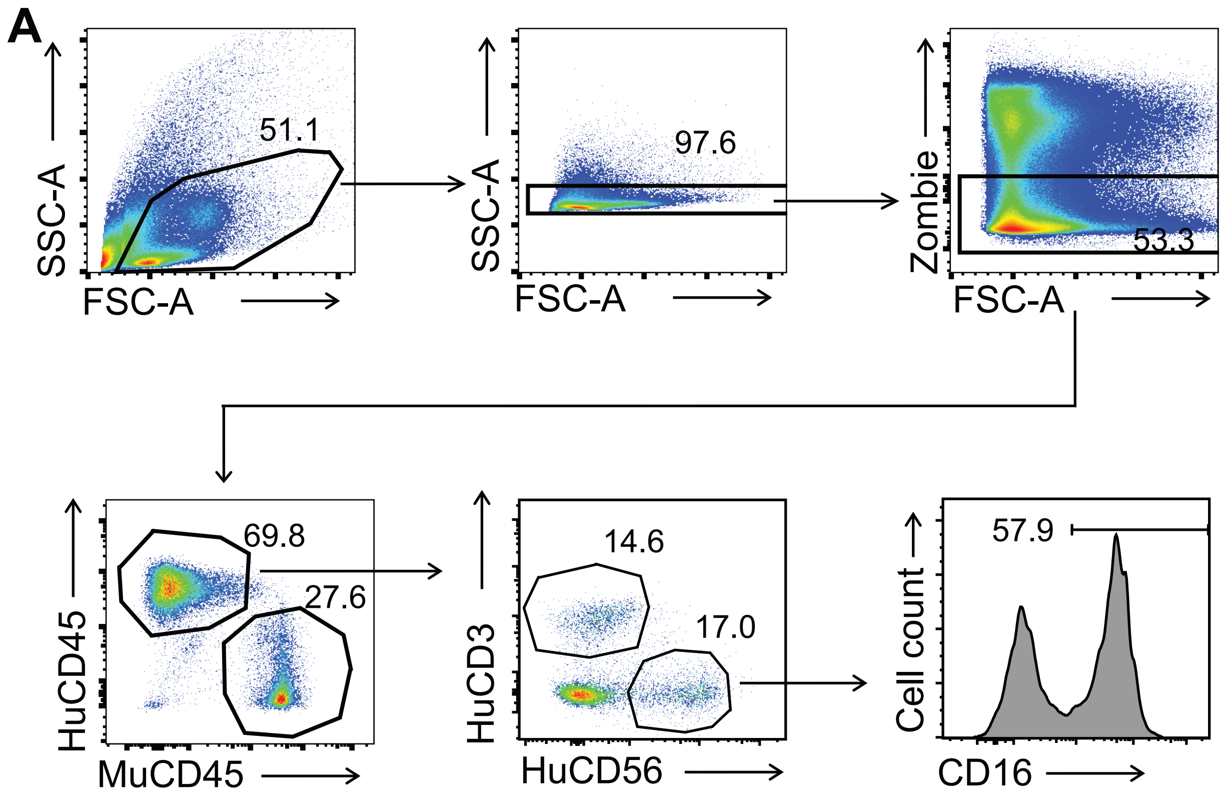

A
51.1
SSC-A
FSC-A
97.6
SSC-A
FSC-A
Zombie
FSC-A
53.3
69.8
27.6
HuCD45
MuCD45
14.6
17.0
HuCD3
HuCD56
57.9
Cell count
CD16

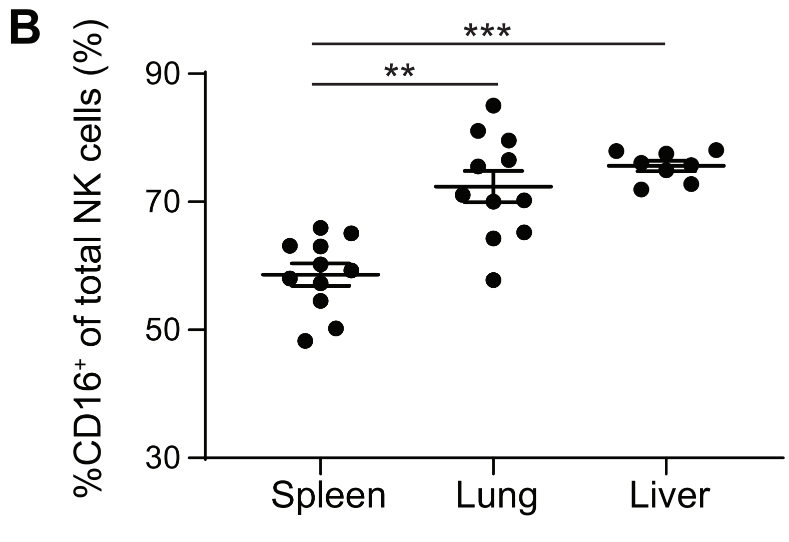

B
***
**
90
70
50
30
%CD16+ of total NK cells (%)
Spleen
Lung
Liver

Supplemental Fig. 5. $CD16^+$ NK cells in MISTRG-6-15 mice.

A, Human NK cell gating strategy. Total splenocytes were used. **B**, percentage of $CD16^+$ NK cells in spleen, lung and liver. Error bars show mean values with SEM. *P*-values were calculated using a one-way ANOVA with Tukey multi-comparison post-test. ** denotes p-value <0.01 and *** denotes p-value <0.001.

Figure S6

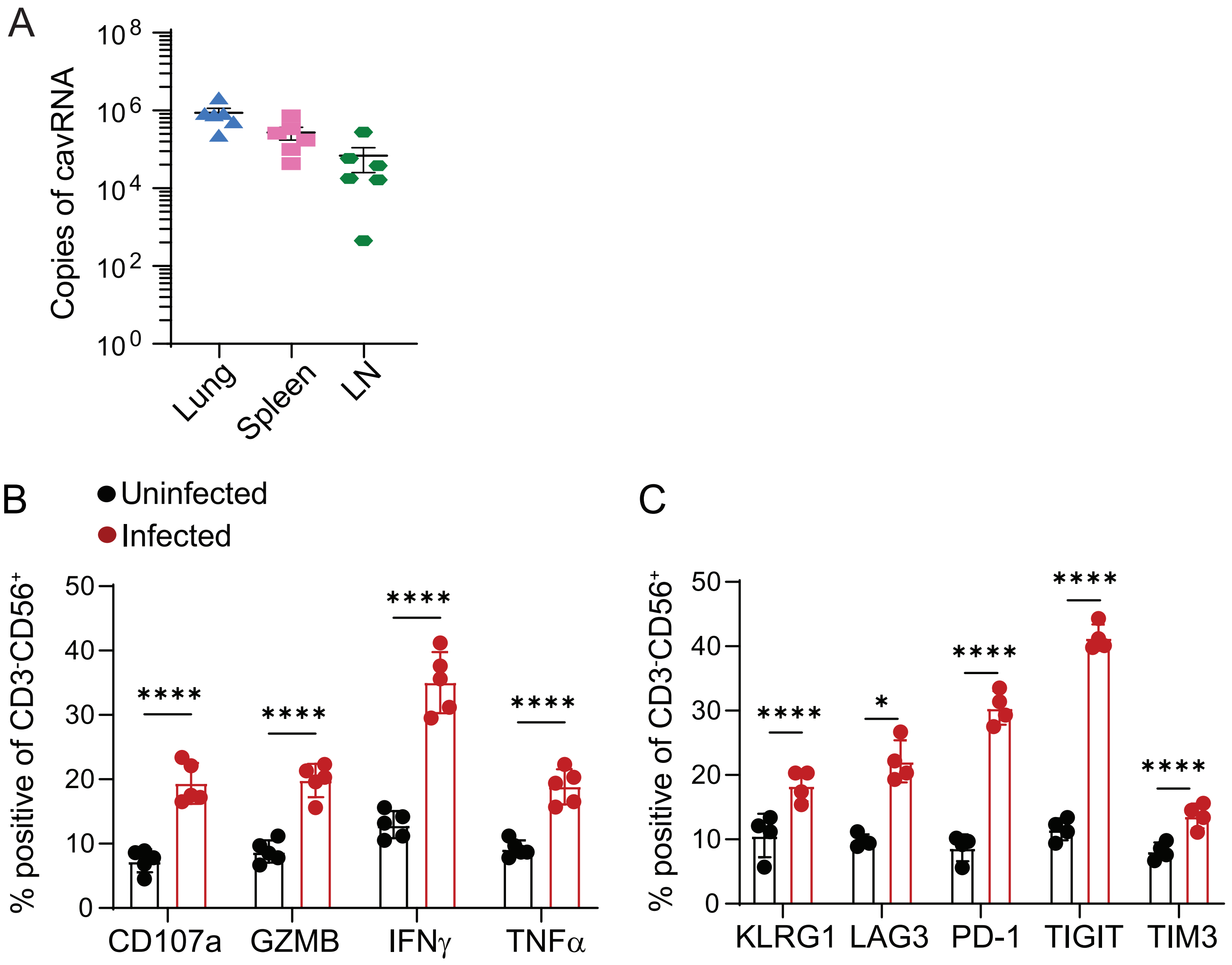
A
Copies of cavRNA
10^8
10^6
10^4
10^2
10^0
Lung
Spleen
LN
B
Uninfected
Infected
% positive of CD3-CD56+
50
40
30
20
10
0
****
****
****
****
CD107a
GZMB
IFNγ
TNFα
C
% positive of CD3-CD56+
50
40
30
20
10
0
****
*
****
****
****
KLRG1
LAG3
PD-1
TIGIT
TIM3

Supplemental Fig. 6. HIV-1 infection and blood NK responses. MISTRG-6-15 mice were infected with HIV-1_{BAL}. Blood and tissues were collected on day 21 post infection. **A,** Copies of cell-associated viral RNA (cavRNA) in lung, spleen and LN. **B**, Percentage of blood NK cells positive for CD107a, GZMB, IFNγ, and TNFα after ex vivo stimulation with PMA/ionomycin for 4 hours. **C**, Percentage of blood NK cells positive for KLRG1, LAG3, PD-1, TIGIT, and TIM3. Error bars show mean values with SEM. *P*-values were calculated using two-way ANOVA with Sidak's multiple comparison test. * denotes p-value <0.05, and **** denotes p-value <0.0001.

Figure S7

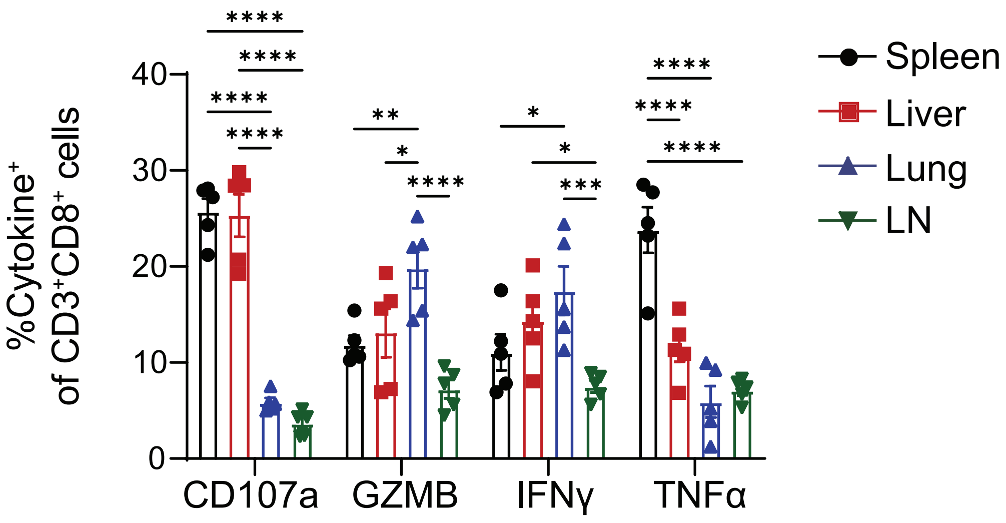

%Cytokine+ of CD3+CD8+ cells
0
10
20
30
40
CD107a
GZMB
IFNγ
TNFα
Spleen
Liver
Lung
LN
****
**
*
***

Supplemental Fig. 7. Functionality of $CD8^+$ T cells during HIV-1 infection in different organs.

MISTRG-6-15 mice were infected with HIV-1_{BAL}. Tissues were collected on day 21 post infection. Percentage of $CD8^+$ T cells positive for either CD107a, granzyme B, IFNγ, or TNFα in spleen, liver, lung, and lymph nodes after ex vivo stimulation with PMA/ionomycin for 4 hours. $CD8^+$ T cells were gated on $huCD45^+CD3^+CD8^+$. Error bars show mean values with SEM. *P*-values were calculated using two-way ANOVA with Tukey multi-comparison post-test. * denotes p-value <0.05, ** denotes p-value <0.01, *** denotes p-value <0.001, and **** denotes p-value <0.0001.

Figure S8

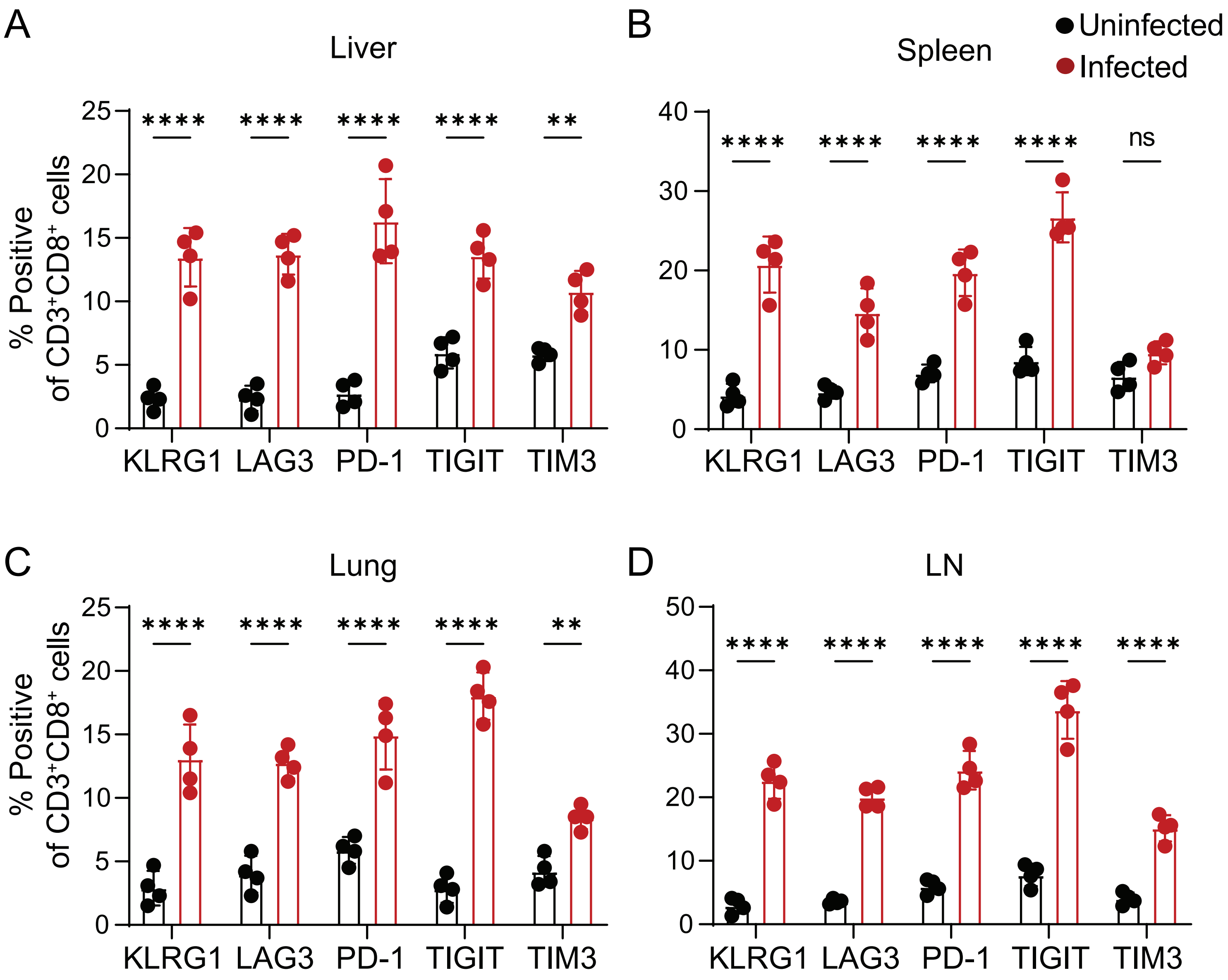
A
Liver
B
Spleen
Uninfected
Infected
% Positive of CD3+CD8+ cells
25
20
15
10
5
0
40
30
20
10
0
****
**
ns
KLRG1
LAG3
PD-1
TIGIT
TIM3
C
Lung
D
LN
50

Supplemental Fig. 8. ICR expression in $CD8^+$ T cells during HIV-1 infection in different organs.

MISTRG-6-15 mice were infected with HIV-1_{BAL}. Tissues were collected on day 21 post infection. Percentage of tissue $CD8^+$ T cells positive for KLRG1, LAG3, PD-1, TIGIT, and TIM3 in liver, spleen, lung, and lymph nodes. $CD8^+$ T cells were gated on $huCD45^+CD3^+CD8^+$. Error bars show mean values with SEM. *P*-values were calculated using two-way ANOVA with Sidak's multiple comparison test. ** denotes p-value <0.01, and **** denotes p-value <0.0001.

Figure S9

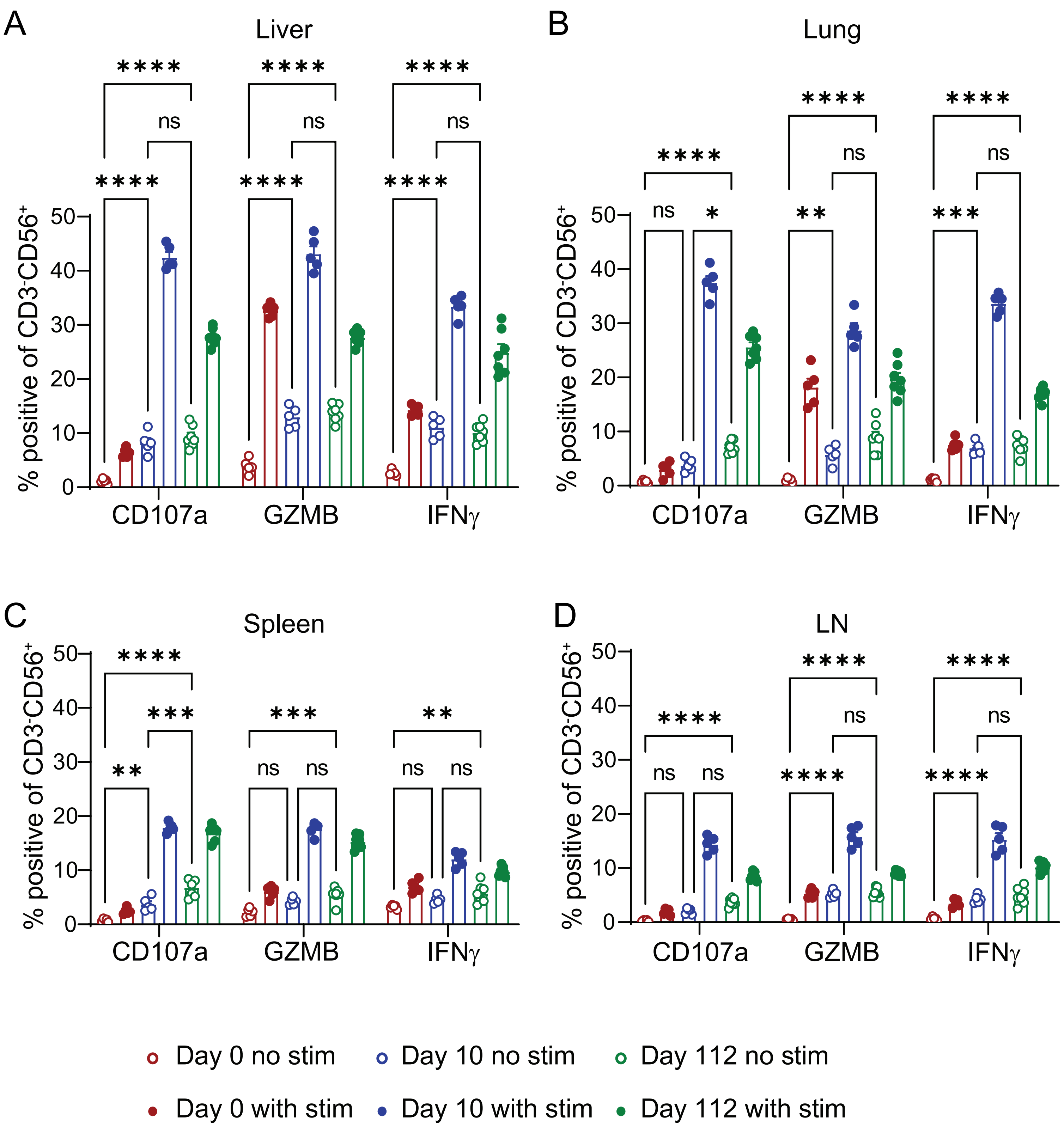
A
Liver
B
Lung
C
Spleen
D
LN
% positive of CD3-CD56+
CD107a
GZMB
IFNγ
Day 0 no stim
Day 10 no stim
Day 112 no stim
Day 0 with stim
Day 10 with stim
Day 112 with stim

Supplemental Fig. 9. NK cell response throughout the course of HIV-1 infection. MISTRG-6-15 mice were infected with HIV-1_{BAL}. Tissue analyses were performed on day 0, 10, and 112 post infection. 4-6 mice at each time point were included. Percentage of $CD107a^{+}$, $GZMB^{+}$, and $IFN\gamma^{+}$ NK cells in liver, lung, spleen, and LN with or without ex vivo stimulation with PMA/ionomycin for 4 hours. NK cells were gated on $huCD45^{+}CD3^{-}CD56^{+}$. Error bars show mean values with SEM. *P*-values were calculated using two-way ANOVA with Tukey multi-comparison post-test. ** denotes p-value <0.01, *** denotes p-value <0.001, and **** denotes p-value <0.0001.

Figure S10

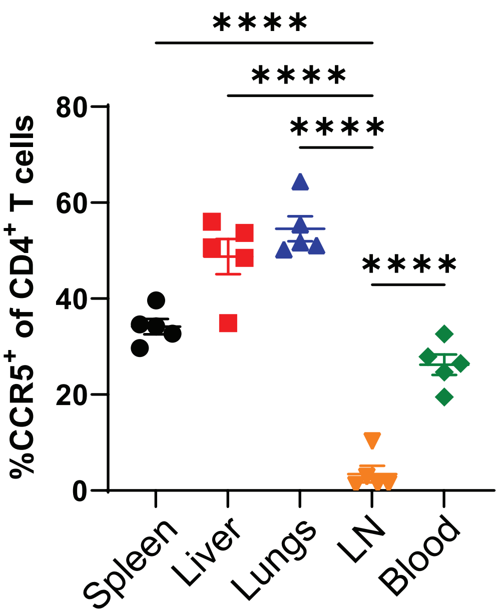

Supplemental Fig. 10. $CCR5^+CD4^+$ T cells in tissues.

Percentage of $CCR5^+$ of total $CD4^+$ T cells in spleen, liver, lung, LN, and blood of MISTRG-6-15 mice. MISTRG-6-15 mice were infected with HIV-1_{BAL}. Tissue analyses were performed on day 14 post infection. Error bars show mean values with SEM. *P*-values were calculated using one-way ANOVA with Tukey multi-comparison post-test. **** denotes p-value <0.0001.

Figure S11

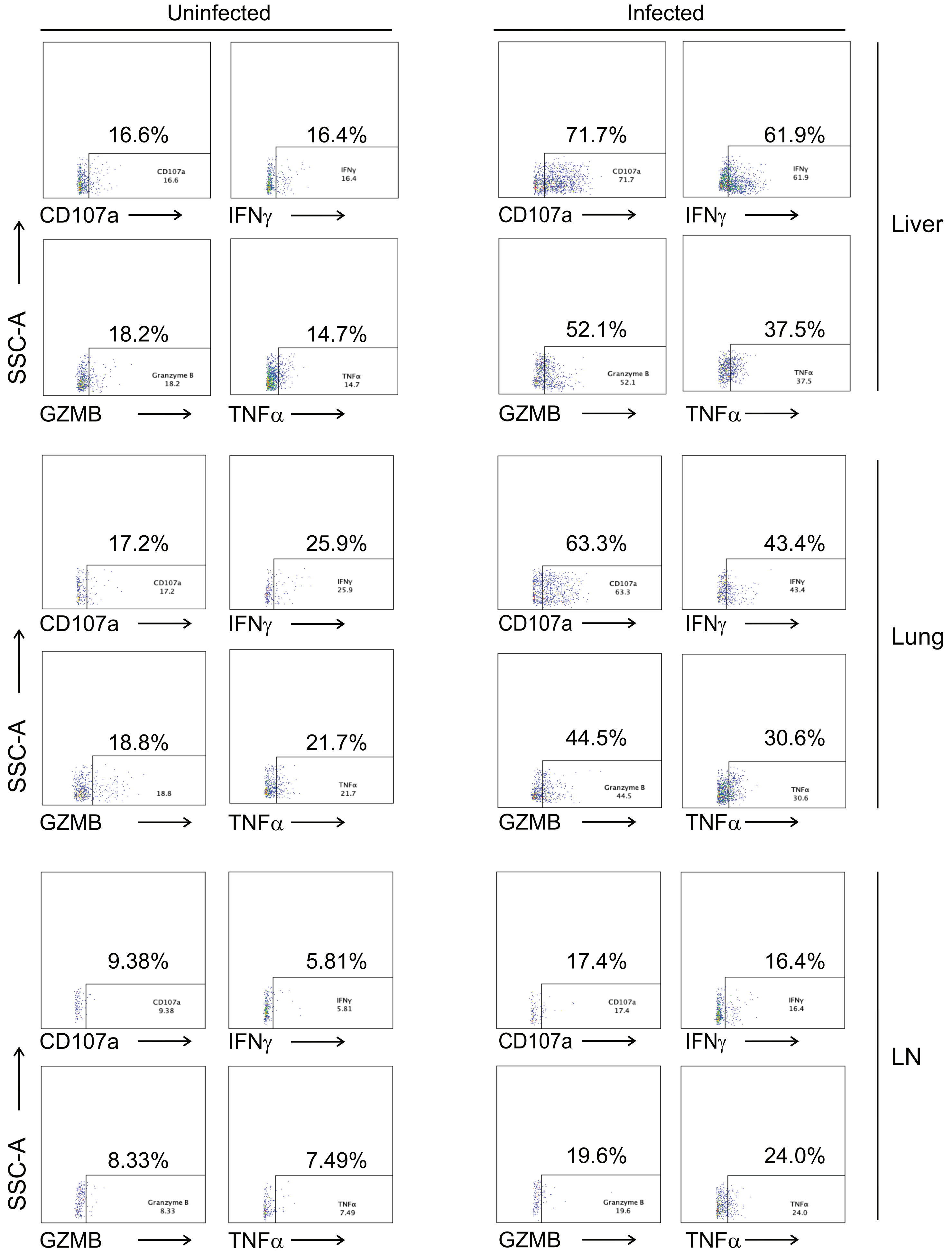

Uninfected
Infected
16.6%
CD107a 16.6
16.4%
IFNγ 16.4
71.7%
CD107a 71.7
61.9%
IFNγ 61.9
CD107a
IFNγ
Liver
SSC-A
18.2%
Granzyme B 18.2
14.7%
TNFα 14.7
52.1%
Granzyme B 52.1
37.5%
TNFα 37.5
GZMB
TNFα
17.2%
CD107a 17.2
25.9%
IFNγ 25.9
63.3%
CD107a 63.3
43.4%
IFNγ 43.4
Lung
18.8%
18.8
21.7%
TNFα 21.7
44.5%
Granzyme B 44.5
30.6%
TNFα 30.6
9.38%
CD107a 9.38
5.81%
IFNγ 5.81
17.4%
CD107a 17.4
16.4%
IFNγ 16.4
LN
8.33%
Granzyme B 8.33
7.49%
TNFα 7.49
19.6%
Granzyme B 19.6
24.0%
TNFα 24.0

Supplemental Fig. 11. NK cell cytokine production by intracellular staining. MISTRG-6-15 mice were infected with HIV-1_{BAL} or left uninfected. Tissue samples were collected on day 21 post infection. Representative flow plots for CD107a, GZMB, IFNγ, and TNFα staining after ex vivo stimulation with PMA/ionomycin for 4 hrs. NK cells were gated on $huCD45^{+}CD3^{-}CD56^{+}$.
